# Modelling Hair Follicle Growth Dynamics as an Excitable Medium

**DOI:** 10.1371/journal.pcbi.1002804

**Published:** 2012-12-20

**Authors:** Philip J. Murray, Philip K. Maini, Maksim V. Plikus, Cheng-Ming Chuong, Ruth E. Baker

**Affiliations:** 1 Centre for Mathematical Biology, Mathematical Institute, Oxford, United Kingdom; 2 Oxford Centre for Integrative Systems Biology, Department of Biochemistry, Oxford, United Kingdom; 3 Department of Developmental and Cell Biology, Sue and Bill Gross Stem Cell Research Center, Irvine, California, United States of America; 4 Department of Pathology, University of Southern California, Los Angeles, California, United States of America; Princeton University, United States of America

## Abstract

The hair follicle system represents a tractable model for the study of stem cell behaviour in regenerative adult epithelial tissue. However, although there are numerous spatial scales of observation (molecular, cellular, follicle and multi follicle), it is not yet clear what mechanisms underpin the follicle growth cycle. In this study we seek to address this problem by describing how the growth dynamics of a large population of follicles can be treated as a classical excitable medium. Defining caricature interactions at the molecular scale and treating a single follicle as a functional unit, a minimal model is proposed in which the follicle growth cycle is an emergent phenomenon. Expressions are derived, in terms of parameters representing molecular regulation, for the time spent in the different functional phases of the cycle, a formalism that allows the model to be directly compared with a previous cellular automaton model and experimental measurements made at the single follicle scale. A multi follicle model is constructed and numerical simulations are used to demonstrate excellent qualitative agreement with a range of experimental observations. Notably, the excitable medium equations exhibit a wider family of solutions than the previous work and we demonstrate how parameter changes representing altered molecular regulation can explain perturbed patterns in Wnt over-expression and BMP down-regulation mouse models. Further experimental scenarios that could be used to test the fundamental premise of the model are suggested. The key conclusion from our work is that positive and negative regulatory interactions between activators and inhibitors can give rise to a range of experimentally observed phenomena at the follicle and multi follicle spatial scales and, as such, could represent a core mechanism underlying hair follicle growth.

## Author Summary

Although the molecular interactions that regulate the follicle growth cycle have begun to be uncovered, the fundamental interactions that regulate periodicity remain elusive. In this study we develop a model in which we neglect biophysical effects (and hence morphological changes) by treating each follicle as a functional unit. We then describe caricature interactions at the follicle scale which have the property that a field of coupled follicles can be treated as an excitable medium. We perform a range of simulations that demonstrate qualitative agreement with experimental observations. Furthermore, the modelling results suggest a regulatory mechanism that might represent a key underlying principle in the regulation of hair growth.

## Introduction

Hair is a characteristic feature of mammals and performs a variety of roles, such as thermal insulation, physical protection, camouflage, social interaction and sensory perception [1]. The relative importance of the different functions of hair depend on a host of factors (e.g. local environment) and it is often crucial that an individual can adapt its coat accordingly. Such control is perhaps most evident in the periodic shedding of fur in response to seasonal changes [2].

The base of a hair resides in an approximately cylindrically shaped, multicellular mini-organ called a hair follicle that is invaginated in the surface of the skin. Unlike the hair itself, which is composed of dead keratinocytes, hair follicles undergo a process of cyclical regeneration, regulated by an intrinsic clock as well as other extrinsic mechanisms [2], that allows for the localised growth of individual hairs. The inner surface of the follicle is lined by epithelial cells and its rate of regeneration is ultimately controlled by the rate at which follicle stem cells exit their quiescent state and become activated.

**Figure 1:**
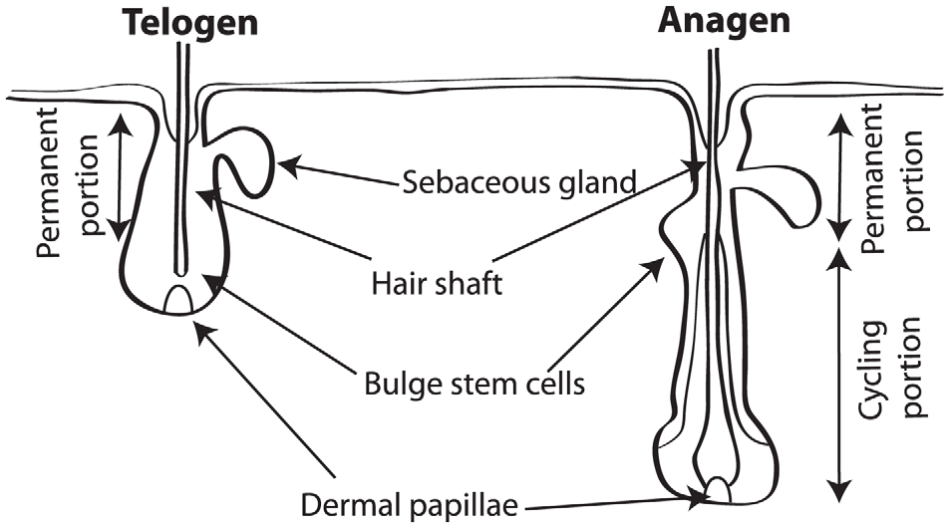
A schematic illustration of the morphological changes that a follicle undergoes in the transition from telogen to anagen (and *vice-versa*).

The follicle growth cycle is traditionally split into three phases: anagen and catagen, when growth and involution occur, respectively, and telogen, a quiescent phase when the follicle is either refractory or awaiting re-entry into anagen [1], [3]. A follicle undergoes substantial morphological changes as the cycle progresses (see Figure 1): during telogen, the dermal papilla, a mesenchymal tissue at the proximal end of the follicle, is in close proximity to a stem cell niche that resides in a spatial region known as the follicle bulge. Upon anagen entry, stem cells in the bulge proliferate and generate transit amplifying cells, and the proximal end of the follicle (including the dermal papilla) extends proximally. As anagen progresses the transit cells differentiate and form the new hair shaft. Transition to catagen results in a rapid bout of apoptosis, the proximal end of the follicle involutes and the dermal papilla returns again to a position in close proximity to the follicle bulge. During telogen the morphological features of the follicle remain relatively conserved.

**Figure 2:**
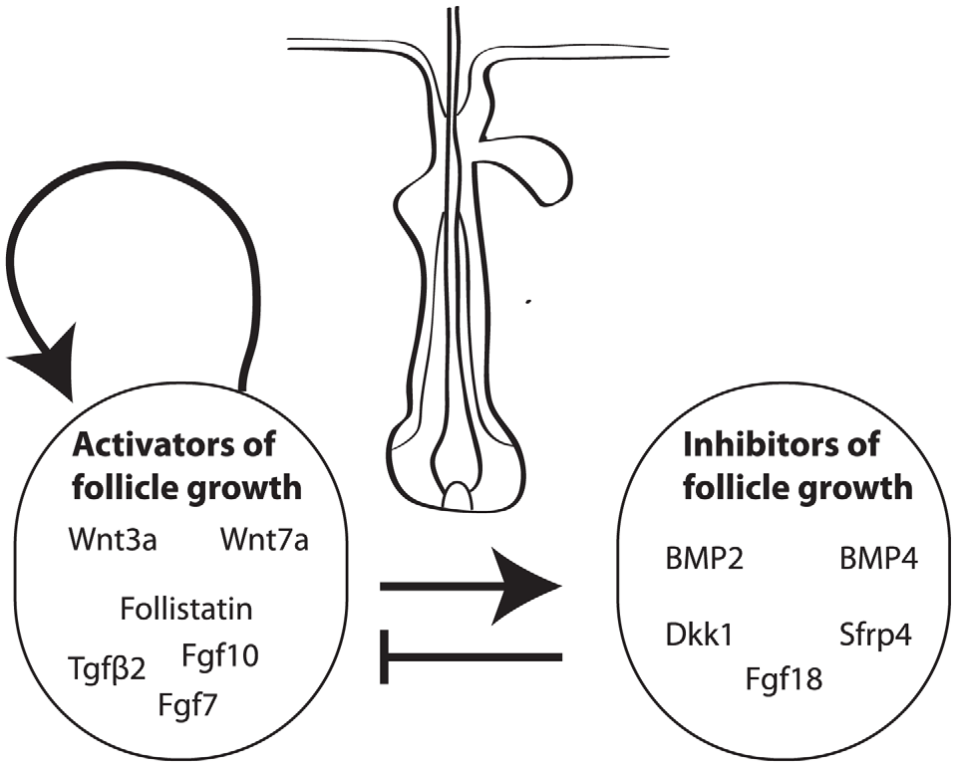
A schematic illustration of known activators and inhibitors of follicle growth.

**Figure 3:**
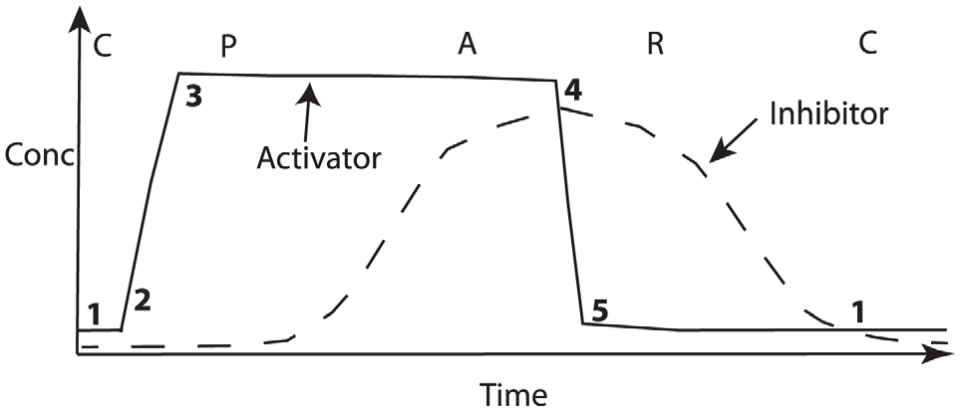
A schematic illustration of the PARC model. The functional phases, P (propagating anagen), A (autonomous anagen), R (refractory telogen) and C (competent telogen) are plotted alongside activator and inhibitor concentrations as a follicle proceeds through a single cycle of the clock. 1,..,5 denote notable transition points in the activator/inhibitor trajectories and are included to aid comparison with the corresponding phase plane diagram presented in Figure 5.

Although it has been established that the hair follicle clock is controlled by interactions local to the hair follicle [2]–[5] and a large number of different extrafollicular signals (*e.g.* hormones, neuropeptides, growth factors) are known to impact upon follicle growth (see Figure 2), the fundamental interactions underlying the follicle clock remain elusive [1], [2], [6]–[9]. However, specific molecular pathways that become activated in different phases of the follicle cycle have been identified (BMP, Wnt, Fgf and TGF 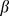) and have been shown to, at least partially, control follicle growth dynamics [10]. For instance, using the transgenic mice *KRT14-Wnt7a* and *KRT14-Nog*, the Wnt and BMP pathways have been identified as activators and inhibitors of localised follicle growth, respectively [11]. These results were further corroborated using coated bead implants in wild-type mice. Notably, the Wnt and BMP pathways cycle out of phase with one another, with BMP activity high during refractory telogen and Wnt during anagen [11] (see schematic diagram in Figure 3). Whilst the close correlation between anagen/telogen and Wnt/BMP pathway activity has led to speculation that interaction between the Wnt and BMP pathways might provide a potential mechanism that governs the follicle cycle [8], recent observations in which members of the Fgf and TGF 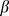 signalling pathways have also been shown to perturb follicle growth suggest that regulation of the hair follicle cycle in mouse is mediated via multiple different molecular pathways.

As illustrated in Figure 1, a follicle has a number of physically distinct regions that can influence the proliferation of stem cells in the follicle bulge. For instance, the dermal papilla is a source of Wnt ligands but it is also maintained in anagen by *Wnt3a* and *Wnt7a* ligands [4]. Furthermore, when stabilized 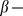
catenin is artificially elevated in resting stem cells, hair follicles are precociously induced to begin a new round of hair growth [8], [12]–[14]. In contrast, cyclic BMP expression has been observed in adipocytes that reside in extrafollicular space [11] and it is thought that high levels of BMP signalling can maintain bulge stem cells in a quiescent state during telogen. For instance, Kobielak et al. have shown, via conditional ablation of a BMP receptor gene, that the BMP pathway inhibits the initiation of the hair cycle [15]. Moreover, BMP activity is stimulated by anagen progression which itself is stimulated by activator expression. Combined, these observations are suggestive of dermal papilla/Wnt mediated positive feedback signalling that results in the activation of stem cell proliferation in the bulge stem cell region and hence follicle growth, and a negative feedback loop in which anagen-inducing activators cause, perhaps indirectly, the production of telogen-inducing inhibitors, which themselves inhibit the activators of follicle growth. We again highlight that, although the influence of particular activators and inhibitors on hair growth is beginning to become better understood, precisely how the activators and inhibitors of follicle growth interact with one another has not yet been well characterised.

**Figure 4:**
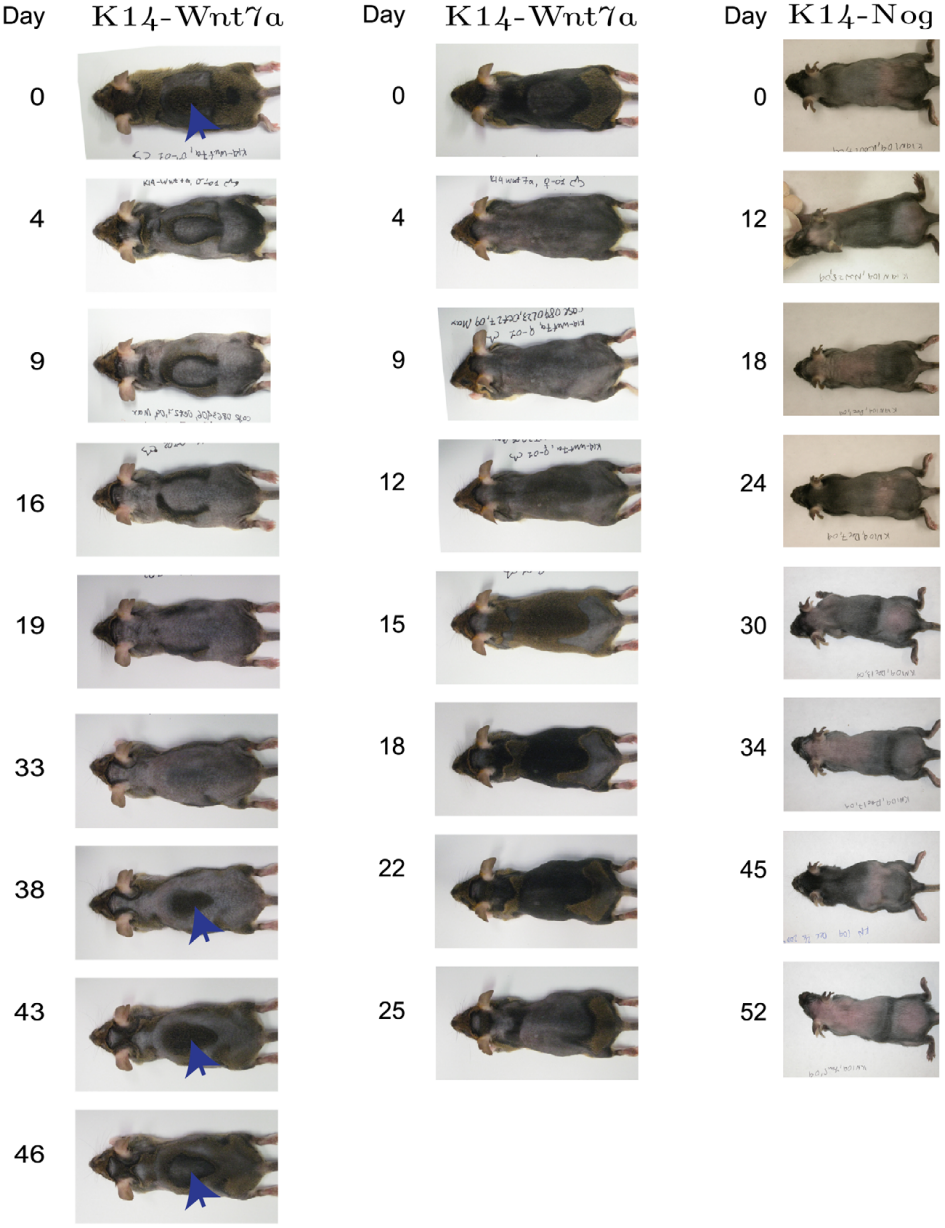
Macroscale patterns observed in *K14-Wnt7a* and *K14-Nog* over-expression mice. Left: target-like patterns of hair growth arise in the *K14-Wnt7a* over-expression mouse. Arrows denote central region of target patterns. Centre: less symmetrical growth patterns also arise in the *K14-Wnt7a* over-expression mouse. Right: Oscillatory patterns in the Noggin over-expression mouse. Similar growth patterns can be found in the Supplementary Material of [16].

At the multi follicle scale, it has been observed that follicles can either make the transition from telogen to anagen autonomously or via induction by neighbouring follicles that have themselves just entered anagen [11]. The former mechanism introduces stochasticity into follicle growth dynamics, as is evident from the random initiation sites of hair growth observed *in vivo* [11]. The latter mechanism allows coordinated behaviour amongst populations of follicles, resulting in the propagation of waves of hair growth (see Figure 4), and is thought to be mediated by the diffusion of activators and/or inhibitors [16]. Furthermore, long range signalling has been demonstrated in experiments where beads coated in activators/inhibitors have been shown to promote/inhibit follicle growth over extended spatial distances [11].

Whilst the growth cycle of a single hair follicle is dependent on coupled physical (*e.g.* cell movement), biochemical (multiple pathways) and biological (*e.g.* apoptosis and cell proliferation) processes, the treatment of a single follicle as a functional unit has allowed Plikus et al. [11], [16] to probe the nature of the follicle cycle in a ‘top down’ manner. For instance, quantification of the cycle resulted in the proposal that there are four functional stages in the follicle growth cycle: propagating anagen (P), when a follicle can induce neighbouring follicles in telogen to enter anagen; autonomous anagen (A), when follicles can no longer communicate with their neighbours but are still in the growth phase; refractory telogen (R), when a follicle is no longer undergoing growth and neither influences nor is influenced by its neighbours; and competent telogen (C), when a follicle can either enter anagen spontaneously or be induced to do so by neighbours in propagating anagen. The times individual follicles spend in the different phases of the hair cycle have been measured (see Table 1) and these data used to parameterise a phase-structured model (which will be denoted by PARC) of the follicle growth cycle.

**Table 1:** A table of measurements used to parameterise the PARC phase structured model of a single follicle.

Mouse model	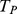	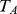	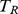	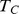	Units	Reference
Wild-type	4	10	28	0–60	d	[11]
*KRT14-Wnt 7a*	14	0	12	0–15	d	[16]
*KRT14-Nog*	4	10	6	0–5	d	[11]

As well as quantifying the excitable dynamics of individual follicles, Plikus et al. have exploited the coupling between anagen and the production of pigmentation [7] in order to obtain a spatial readout of the temporal dynamics of follicle activity (the pigment is macroscopically observable on the surface of a clipped animal thus giving a readout of which follicles are in, or have recently been in, anagen). The results from such follicle population scale experiments can be seen in Figure 4 where the follicle growth patterns observed in Wnt over-expression (*KRT14-Wnt7a*) and BMP down-regulation (*KRT14-Nog*) mice models are compared.

Simulations at the multi follicle scale have previously been modelled using cellular automata [11], [16]–[18]. Halloy et al. [17], [18], considering human hair growth dynamics, originally proposed a follicular automaton model in which measurements of the functional phases of the hair cycle were used to parameterise a cellular automaton model. The phenomenon of inter follicle coupling was neglected as it is thought to play a negligible role in human hair growth dynamics. In contrast, communication between neighbouring follicles in mice is well established and, accordingly, Plikus et al. [11], [16] developed a cellular automaton model of mouse follicles that accounted for local coupling between neighbouring follicles. Plikus et al. also used experimental measurements of times spent in different phases of the hair cycle to parameterise the automaton model and simulated how variation in the behaviour of individual follicles was manifest at the population scale. This approach provided a computational architecture in which to relate follicle scale quantities, such as the mean time spent in R phase, to emergent patterns at the population scale, both in individual organisms and across different species. Moreover, the model produced a range of patterns that exhibited many features in common with experimental observations: wave propagation, spontaneous excitation, border stability and instability (under different conditions). When key parameters in the model, such as the probability of spontaneous excitation, were varied, the emergent patterns varied in similar ways to experimental observations.

Whilst previous cellular automaton models of hair follicle growth provided a useful framework in which to integrate various experimental data and investigate hypotheses [11], [16]–[18], their primary limitation is that the automaton rules are chosen to simulate experimental observations and, hence, are not motivated by underlying mechanisms. Moreover, it can be difficult to meaningfully relate the automaton rules to the increasing amount of experimental data becoming available at the molecular scale. The goal of this study is to develop a model that can begin to bridge the three scales of observation (molecular, single follicle and multi follicle) in the hair follicle system. We demonstrate how populations of hair follicles can be described using a classical excitable medium framework [19], with the mechanisms that control certain features of the follicle dynamics, such as the excitability threshold and length of the different phases in the PARC model, related to regulation by activators and inhibitors of follicle growth. The layout is as follows: firstly, we briefly introduce the well-established theory of excitable media and describe a minimal model of the hair follicle system; secondly, we present simulation results and compare them with experimental observations; thirdly, we describe how a number of model predictions could be tested experimentally; and, finally, we conclude with a summary and discussion.

## Methods

### Introduction to excitable medium dynamics

**Figure 5:**
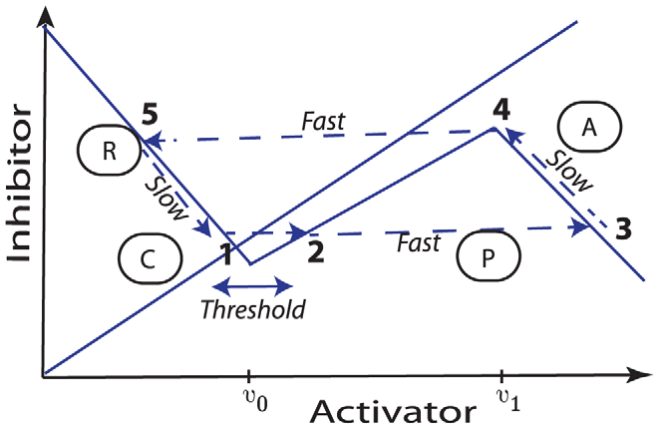
A schematic illustration of an excitable medium. The intersection of the solid lines determines the steady-state corresponding to low activator and inhibitor activities. Upon excitation over the threshold (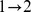), the activator activity increases rapidly (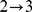). As a consequence, the inhibitor activity then increases on a slow time scale (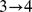). When the inhibitor activity reaches a critical threshold, the activator activity suddenly decreases (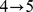). Finally, with low activator levels, the inhibitor activity slowly decreases (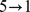) and the system returns to the stable steady-state. P, A, R, and C denote the mapping of the functional phases of the hair follicle cycle onto the excitable medium.

Before discussing the specifics of the hair follicle system, we provide a brief introduction to the theory of excitable media, a field of study that is used to describe a disparate range of fundamental phenomena in biology, such as nerve signal propagation [20], [21], electrical activity in the heart [22], calcium dynamics [23] and dictyostelium aggregation [24]. Whilst the underlying chemical/ionic equations for particular systems are often highly nonlinear, the essence of the phenomenon of excitability can be understood using much simpler models. For example, consider a two-variable activator-inhibitor system that has a single stable steady-state (1) where both activator and inhibitor activities are low (see phase plane diagram in Figure 5). Making the further assumption that the activator activity changes on a much faster time scale than that of the inhibitor, a perturbation of sufficient magnitude (1
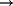
2) can result in the fast activation of the activator and the system moves to a transient state of high activator activity (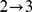). However, a consequence of high activator activity is that the inhibitor slowly gets activated (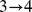) and eventually causes a fast deactivation of the activator (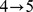). The system remains in a refractory state until the inhibitor activity returns to steady-state levels (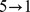), whence the excitable cycle is complete and competent for a further activation.

The central tenet of this study can be described as follows: using an excitable medium framework, a follicle's state is represented by two variables, an activator and an inhibitor of follicle growth. The activator and inhibitor values are correlated with, but not explicitly representative of, the concentrations of known activators and inhibitors of follicle growth, such as members of the Wnt and BMP pathways, respectively. The dynamics of the activators and inhibitors can be described as follows: a follicle has a stable steady-state in which activator and inhibitor activities are low (see Figure 5). The follicle can become excited (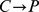), either stochastically or by interaction with neighbours, and activator activity increases on a fast time scale. Activator activity corresponds to anagen so, whilst the activator activity is high, the follicle grows. However, the inhibitor activity increases on a slow time scale (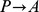) and eventually turns the activator off, thus follicle growth is halted (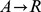). At this stage, inhibitor activity is still high and the follicle is in the refractory phase, *i.e.* it cannot be induced back into the growth cycle. The inhibitor then decays on the slow time scale (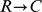) and, eventually, the follicle returns to the competent phase, where upon another growth cycle can be induced upon appropriate perturbation.

But is there experimental evidence in support of the aforementioned hypothesis? At the multi follicle scale, it is clear that patterns of hair follicle growth share many features observed with patterns arising in excitable media (*e.g.* wave propagation, spontaneous excitation, border stability and instability, thresholding). At the individual follicle scale, the functional phases of the hair follicle cycle described by Plikus et al. [11] (propagating anagen, autonomous anagen, refractory telogen, competent telogen) have the properties one expects from an excitable system (excitability, propagation, refractoriness). Moreover, stochasticity in the hair follicle cycle occurs predominantly in competent telogen (see Table 1), which is precisely the behaviour one expects in an excitable system. At the molecular scale the picture is less clear, although the sequence of activations of the Wnt and BMP pathways (see schematic illustration in Figure 3) is consistent with the dynamics of an activator and inhibitor in an excitable medium. Moreover, there is evidence, as described in the introduction, of positive feedback in the Wnt pathway dynamics and negative feedback between BMP and Wnt, interactions that might play a role in the emergence of excitability. In the modelling work that follows we will consider a scale of description at which the follicle is treated as a functional unit and develop a caricature description of activator and inhibitor dynamics at the single follicle scale.

### Model development

#### A single deterministic follicle

**Figure 6:**
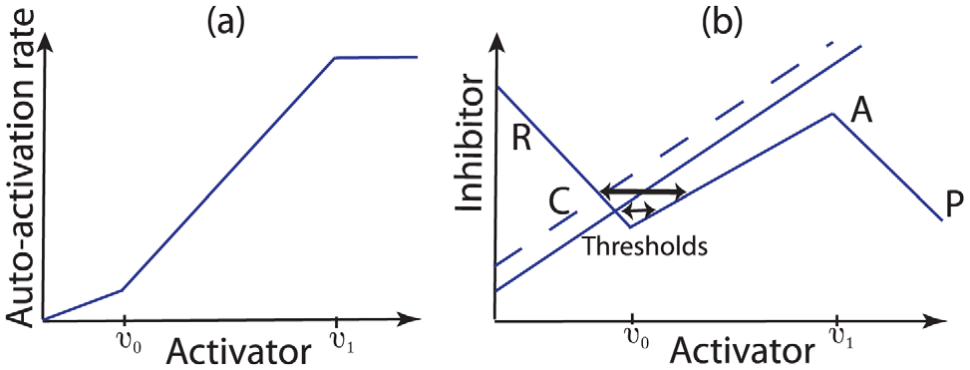
Schematic illustrations of the activator production function and the effect of an increased inhibitor production rate. (a) We assume a switch-like change in auto-activation rate (see equation (3)) as the activator level passes through the threshold values 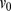 and 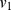. (b) An increase in the inhibitor production rate (dashed line) stabilises the steady-state corresponding to competent telogen.

We assume that the growth of a single follicle is regulated by the activities of an activator, 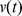, and an inhibitor, 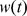, and propose a two variable, FitzHugh-Nagumo-like model [25] with governing equations given by
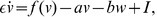
(1)

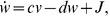
(2)where 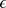 is a small parameter that separates slow and fast time scales in the governing dynamics, 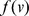 is the activation rate (see Figure 6(a)), 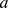 and 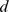 are natural decay rates, 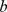 and 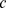 are cross inhibition and activation rates, and 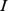 and 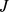 are background rates of activator and inhibitor activation, respectively. The parameters 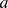, 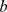, 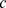, 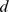, 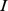 and 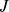 are positive constants.

Equations (1)–(2) have a number of important features that we now highlight. Firstly, the parameter 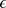 must be small enough such that the activity of the activator changes on a much faster time scale than that of the inhibitor, thus permitting the switching behaviour from anagen to telogen and *vice-versa*, as described in Figure 5 (we later suggest methods by which this assumption could be tested experimentally). Secondly, it is critical that the function 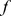 is nonlinear and the simplest caricature case is to let 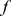 represent a positive feedback loop in the activator dynamics such that the net activation rate 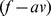 takes the form of a cubic-like function. Thirdly, inhibition of the activator represents the inhibition of activators of follicle growth by inhibitors (e.g. Wnt by BMP), while the activation of the inhibitor represents a slow (perhaps indirect) activation of inhibitors by activators (e.g. BMP by Wnt). Fourthly, the rates 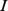 and 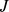 play a critical role in determining how excitable the system is. When 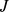 is large (say), there is relatively more inhibitor activity at the steady-state and a larger perturbation is therefore required to excite the system away from equilibrium. Conversely, as 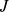 decreases the threshold stimulus required to excite the follicle decreases and, in fact, there is a critical value of 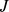 beyond which the excitable behaviour is lost and the follicle becomes oscillatory (see Figure 6(b)). The biological significance of the parameter 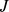 is that it could represent an external mechanism, such as inhibitor production in the subcutaneous tissue, for control of the follicle's excitability.

As the molecular interactions are not yet fully understood in the hair follicle system, in the current study we consider a caricature of excitable dynamics in which the activator dynamics are given by the piece-wise linear function [26] (see schematic in Figure 6(a))
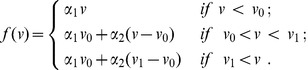
(3)We assume that the constants 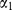, 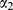, 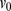 and 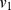 are positive and that the linear activator production rate switches at the threshold activator level 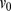 from the rate 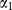 to 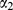. At the threshold activator value 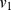, the production attains the maximal rate 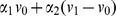. When such production dynamics are coupled with linear decay, the net rate of self-activation takes the form of the cubic nullcline presented in Figure 5 (assuming 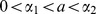).

As the governing equations are linear on the slow time scale, it is straightforward to obtain estimates (see Text S1 for derivations and, for example, [27]) for the time spent on the upwards (anagen) and downwards (refractory) parts of the cycle as
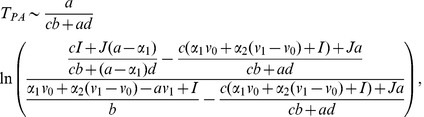
(4)and
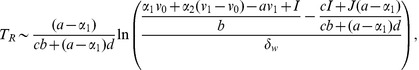
(5)respectively, where 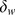 represents a characteristic displacement of 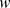 from steady-state (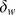 is defined in the following section).

Notably, the simplified description of activator and inhibitor dynamics allows the derivation of times spent in the propagating and refractory phases of the hair cycle, and thus allows the excitable medium description to be directly related to the follicle-scale measurements used in the PARC model. Furthermore, we highlight that the two-variable model is an abstraction in which the variables 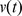 and 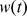 represent the activities of activators and inhibitors of follicle growth, respectively. We expect that the variables 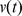 and 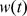 will be correlated with the expression of known activators and inhibitors but note that the variables are not explicitly representative of molecular concentrations. Hence, the negative values attained by 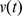 and 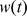 do not violate basic physical principles.

#### A single stochastic follicle

**Figure 7:**
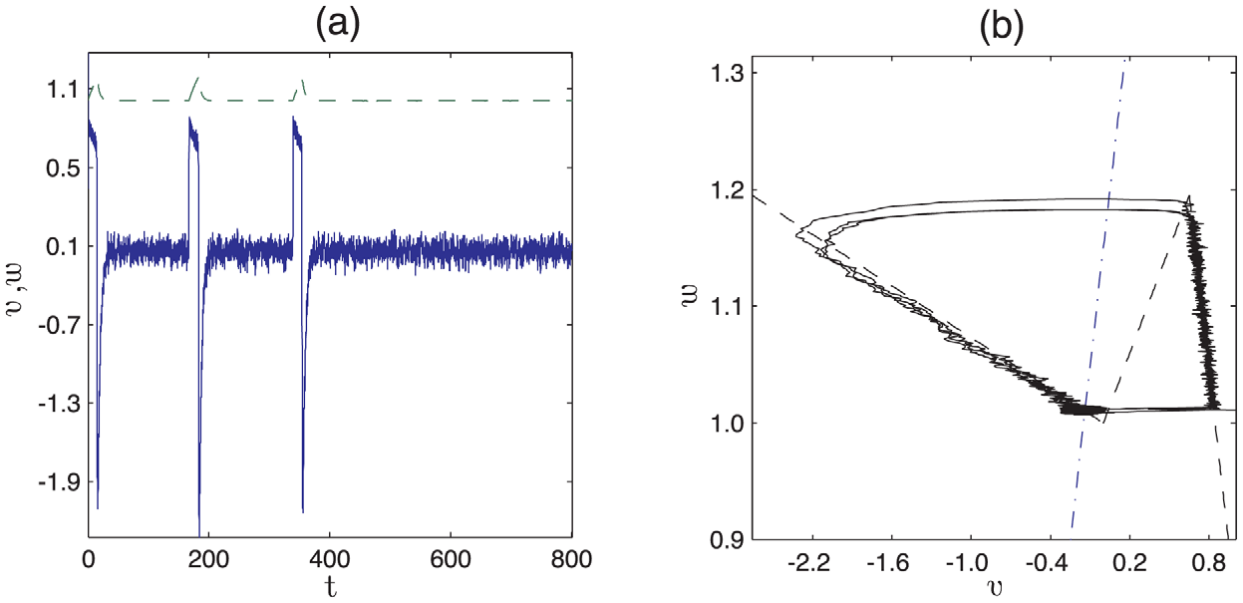
Stochastic firing in the excitable regime. (a) Activator (solid line) and inhibitor (dashed line) activities plotted against time. (b) Trajectories (solid line) and nullclines in 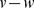 phase space. The nullclines where 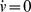 and 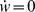 are denoted by dashed and dot-dashed lines, respectively. Equations (6) and (7) were solved with parameters as in Table 2.

**Figure 8:**
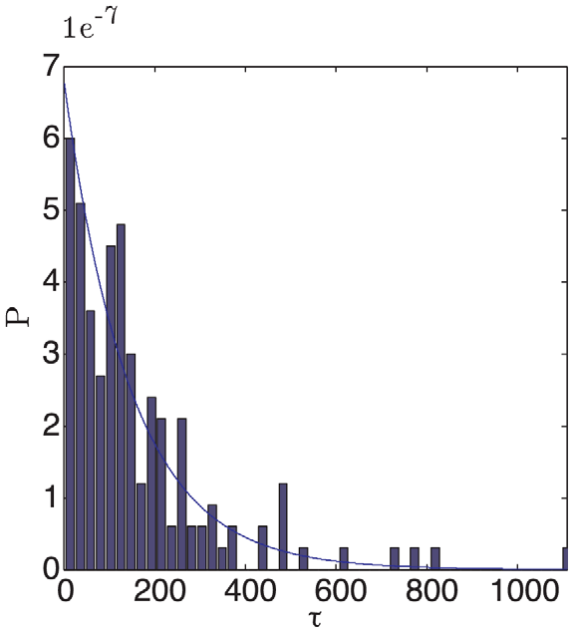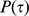, the probability that a follicle spends time 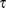 in competent phase, is plotted against 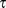. The bars denote measurements of 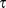 from simulations of equations (6) and (7) with parameters as in Table 2 (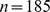). The mean of the distribution, 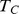, is given by equation (8) while the solid line is the exponential distribution given by equation (9).

Motivated by the *in vivo* observations of new patterning domains emerging at random spatial locations (e.g. [11]), we propose that random fluctuations arising from a variety of factors, such as stochastic behaviour in underlying molecular interactions, can provide the necessary stimulus for a follicle to sporadically enter the growth cycle. We introduce stochasticity into equations (1)–(2) via the inclusion of a Gaussian noise term in the activator dynamics and obtain
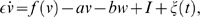
(6)

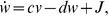
(7)where 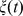 represents a zero-correlated noise defined such that 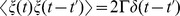. Assuming parameters are chosen such that equations (6)–(7) are in the excitable regime (see Text S1), the noise term can excite the system, resulting in large excursions in phase space that send the follicle into the quasi-steady anagen growth phase before returning to the steady-state (see Figure 7). The relationship between the noise strength and stability of the steady-state strongly influences the effective period of a single follicle. For example, if the noise strength is relatively large then upon exit from refractory telogen, a follicle will almost instantaneously re-enter anagen. In contrast, if the noise strength is relatively small then a follicle will spend a large amount of time at the steady-state awaiting excitation. It can be shown (see Text S1) that in the limit where escape from the steady-state can be treated as a Poisson process, the mean time taken for a follicle to escape from steady-state as a result of stochastic fluctuations (i.e. the mean time spent in competent telogen) is approximately [28]

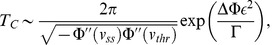
(8)where 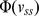, 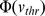 are the effective potential energies when the follicle is in the steady-state and threshold states, respectively, and primes denote differentiation (see Text S1 for derivation). The distribution of escape times then takes the form (see Figure 8)
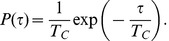
(9)


**Table 2:** A table of parameter values used in the calculation of numerical solutions.

Parameter	Description	Unit	Value
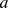	Natural decay rate	d^−1^	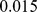
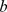	Inhibition rate	d^−1^	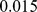
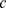	Activation rate	d^−1^	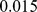
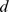	Natural decay rate	d^−1^	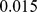
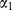	Production rate	d^−1^	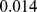
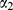	Production rate	d^−1^	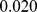
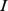	Background activator production rate	d^−1^	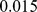
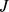	Background inhibitor production rate	d^−1^	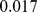
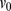	Activator threshold	Nondim	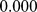
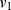	Activator threshold	Nondim	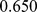
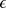	Time scale separation constant	Nondim	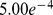
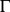	Noise strength	d^−1^	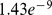
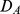	Diffusion coefficient	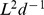	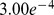
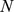	Lattice dimension	Nondim	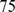

*L* represents inter follicle distance and d days.

The inclusion of stochasticity into the dynamics introduces a noise-dependent characteristic fluctuation for 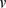 at steady-state that we use to define a boundary between refractory and competent telogen. Hence when the slow variable, 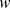, comes within (see Text S1)
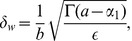
(10)of the (deterministically) stable steady-state value at the end of refractory telogen, we assume that the follicle enters competent telogen. These approximations allow us to estimate 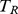 in equation (5). Combining equations (4), (5) and (8), we make the approximation that the mean time for a cell to perform one loop of the excitable trajectory is
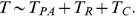
(11)Although the above analysis permits direct correspondence with the PARC model, the key difference between the expression for the period derived in equation (11) and the PARC model is that in the former, the period emerges from an underlying description of the interactions between activators and inhibitors and the system noise. We note also that in the limit of weak noise, stochasticity in the period of the follicle dynamics arises primarily from the time the follicle spends at steady-state awaiting a stochastic fluctuation that allows it to begin an excitable trajectory. This is consistent with the observation that variability in the follicle cycle arises mostly from competent phase (see Table 1). Moreover, in our model the time spent in competent phase (or the waiting-time before excitation) is exponentially distributed (see Figure 8), a prediction that could be verified by a closer investigation of the experimental data used to define the parameters in Table 1.

#### A field of coupled excitable follicles

In order to study populations of interacting follicles on the skin surface, we now consider a two-dimensional field of hair follicles on a regular square 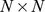 lattice, each of which has the underlying stochastic, excitable dynamics described in the previous section.

It has previously been suggested that the diffusion of activator and/or inhibitor ligands is a potential mechanism for inter-follicular communication [16]. Hence, we account for inter-follicular coupling in equations (6)–(7) via, in the first instance, the introduction of activator diffusion. The governing equations are then given by
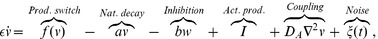
(12)

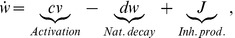
(13)where 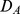 is the activator diffusion coefficient. We note that the inclusion of nearest neighbour diffusive coupling modifies the stability of the steady-state to stochastic perturbations but that this effect is accounted for in a modified derivation of equation (8) in Text S1.

## Results

Using numerical simulations we now demonstrate qualitative agreement between the excitable medium model and a range of experimental observations. Parameters have been chosen such that: (a) the system is in the excitable regime (see Text S1); (b) noise and coupling strengths are sufficiently large so as to excite a follicle; and (c) the times spent in anagen, refractory telogen and competent telogen are in broad agreement with the follicle scale measurements presented in Table 1. We note that although Plikus et al. have measured competent telogen times within a range of 0–60 days, in order for the proposed model to yield stochastic excitations at a rate in agreement with observations we find that 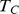 in the model must be of the order of 10^5^ days. We return to this point in the Discussion.

In each of the simulations presented, the governing equations (12) and (13) were solved for a 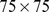 field of follicles in two spatial dimensions using the stochastic Euler-Maruyama method (e.g. [29]) in Matlab. The diffusion term was discretised using a finite difference approximation on a regular square lattice and either periodic or no-flux conditions were imposed on the boundaries of the spatial domain (details for a given simulation are specified in the respective figure caption).

### Simulating observed phenomena in wild-type mice

####  Spontaneous initiation

**Figure 9:**
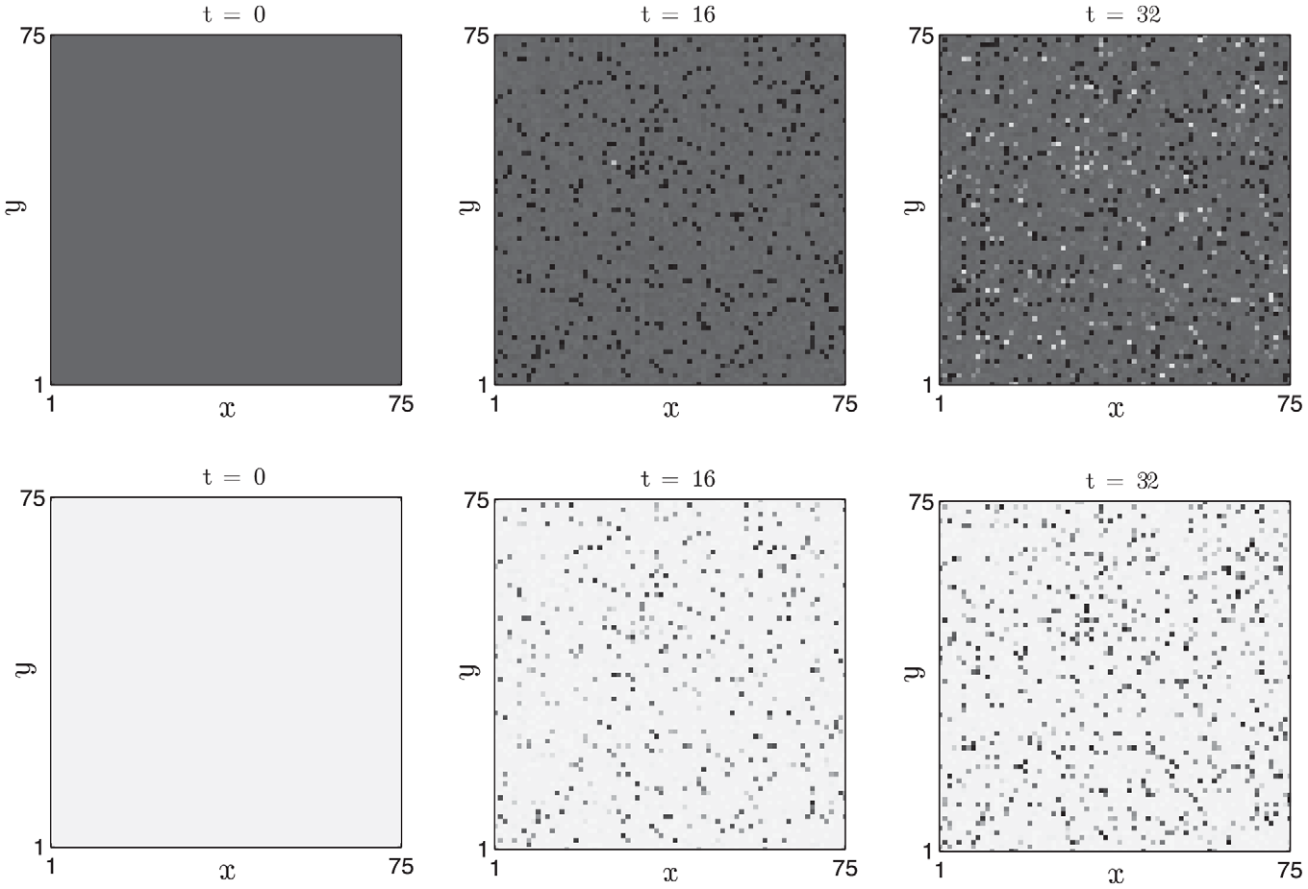
Stochastic activation of anagen in a field of follicles in which diffusive coupling is absent. Activator, 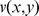, and inhibitor, 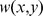, activities (white - low, black - high) are plotted in the top and bottom rows, respectively, at 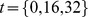 days. Equations (12) and (13) were solved with periodic boundary conditions. 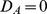 and other parameter values as in Table 2. 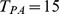, 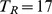, 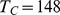 days.

In Figure 9 we present simulation results in which a two-dimensional field of competent follicles undergoes the experimentally observed phenomenon of spontaneous initiations [11], [16]. The diffusive coupling strength is set to zero, hence follicles can only become excited via the autonomous, stochastic mechanism of anagen entry. The noise strength is sufficiently weak such that the stochastic period is dominated by the time spent in competent phase, hence only a small fraction of the follicle population is observed in anagen at a given instant in time.

#### Wave propagation

**Figure 10:**
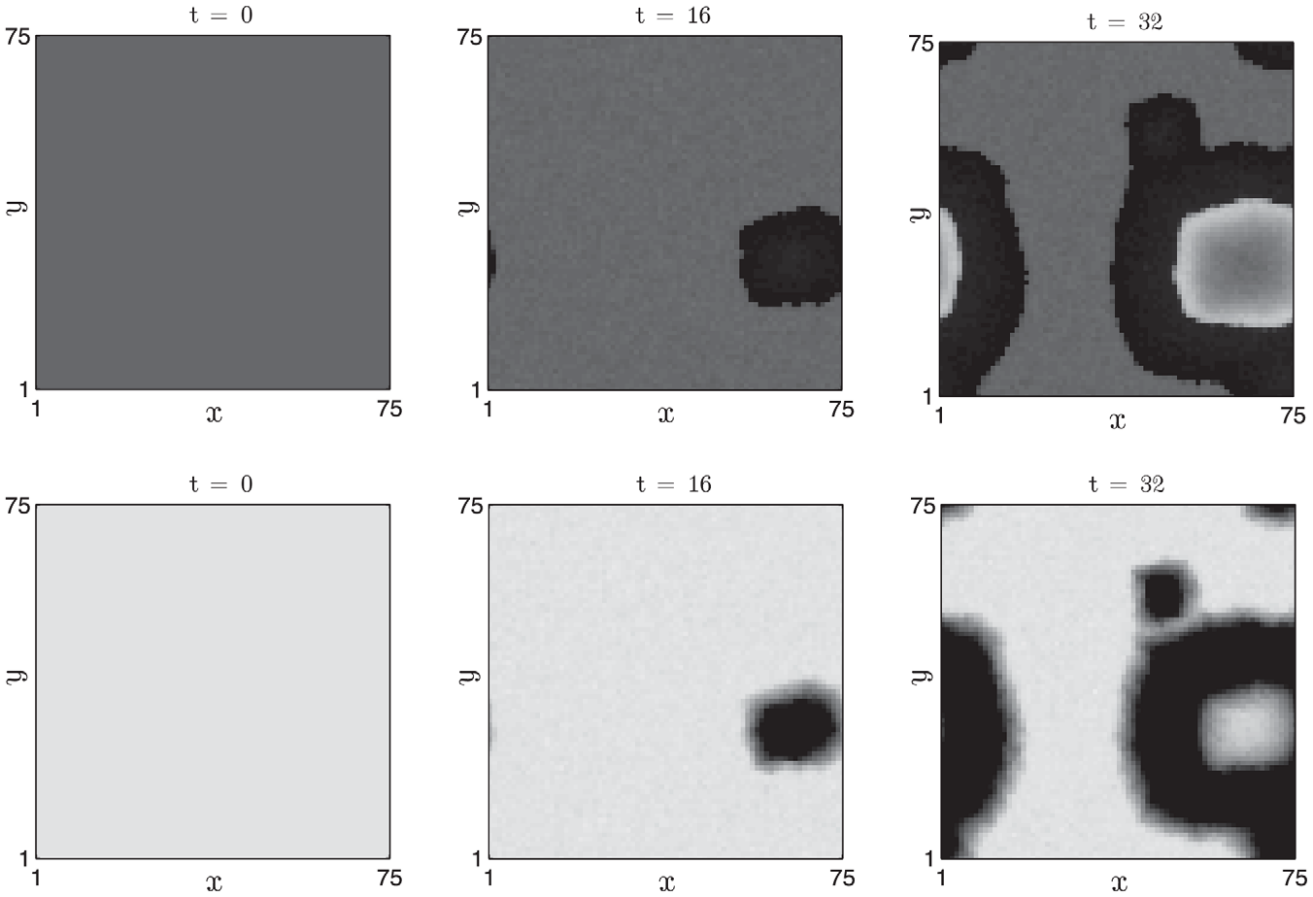
Propagation of single waves after spontaneous activation. Parameter values as in Table 2 (i.e. 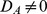) and other details as in Figure 9. 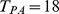, 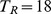, 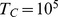 days.

Increasing the coupling strength between neighbouring oscillators, while keeping other parameters fixed, results in propagation of the excited state throughout the follicle field (see Figure 10). The wavefront patterns have simple and predictable temporal dynamics: an excited follicle enters propagating phase (high activator, low inhibitor) and, via the diffusive coupling term, induces neighbouring, competent oscillators to become excited. Subsequently, the local inhibitor activity increases on a slow time scale and eventually deactivates the activator. The follicles then have high inhibitor and low activator activities and are thus refractory. The inhibitor activity decays on the slow time scale and the follicles become competent once more. Notably, the increased diffusion coefficient results in a lower rate of spontaneous activation (compare with Figure 9), an effect that is captured in the approximation for 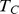 derived in Text S1.

#### Border stability

**Figure 11:**
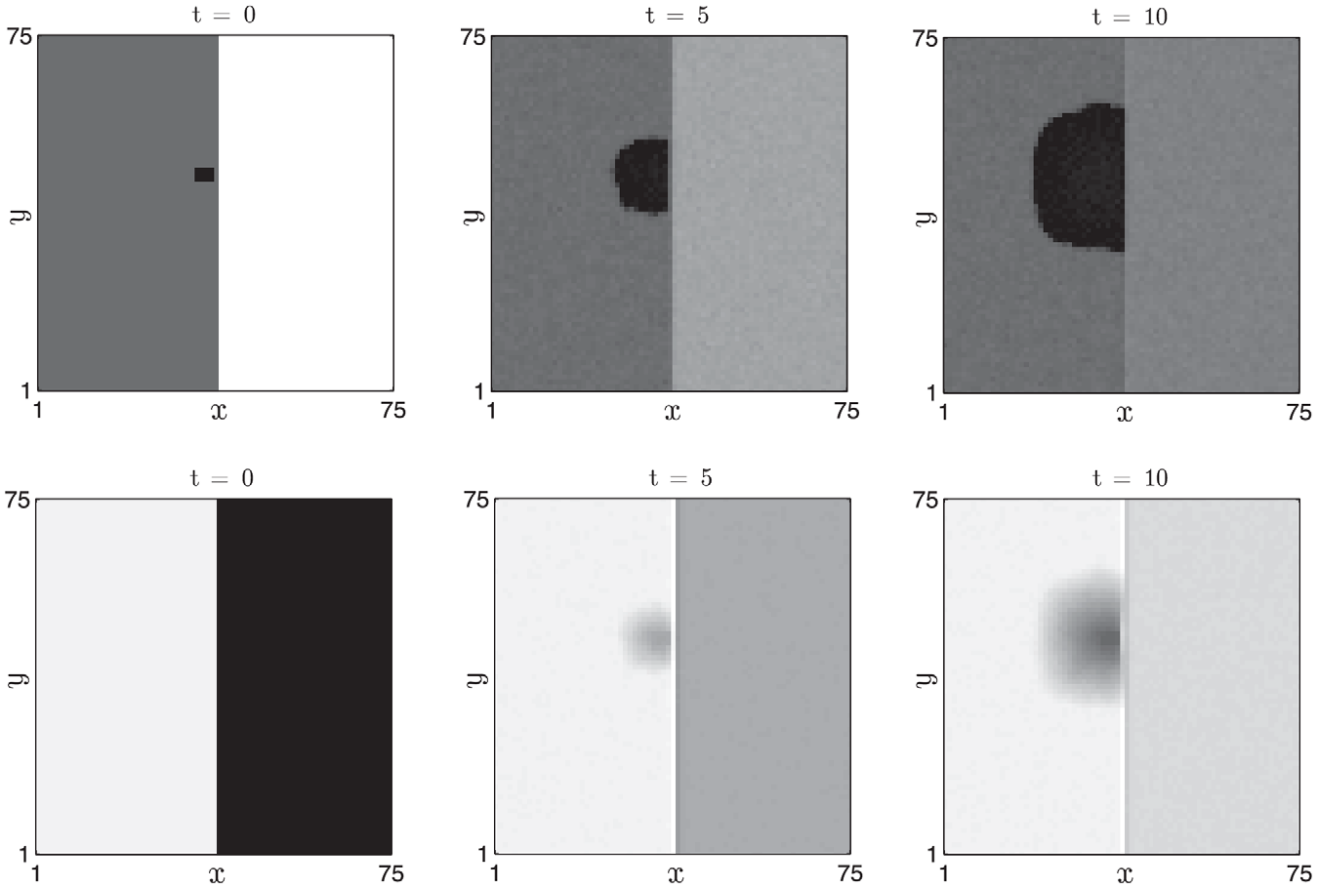
Border stability when the initial data are chosen such that the left- and right- hand sides of the domain are in competent and refractory phases, respectively. Other details as in Figure 10.

We demonstrate border stability by considering initial data in which subpopulations of follicles are in different phases of the cycle. In Figure 11 simulation results are presented in which the left- and right- hand sides of the spatial domain were initially in competent and refractory phases, respectively. At 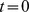 we excite a small region of the competent domain and a wave of propagation ensues. The simulation results clearly indicate that the refractory domain is not penetrated by the wavefront of excited follicles over the time scale of observation.

#### Border instability

**Figure 12:**
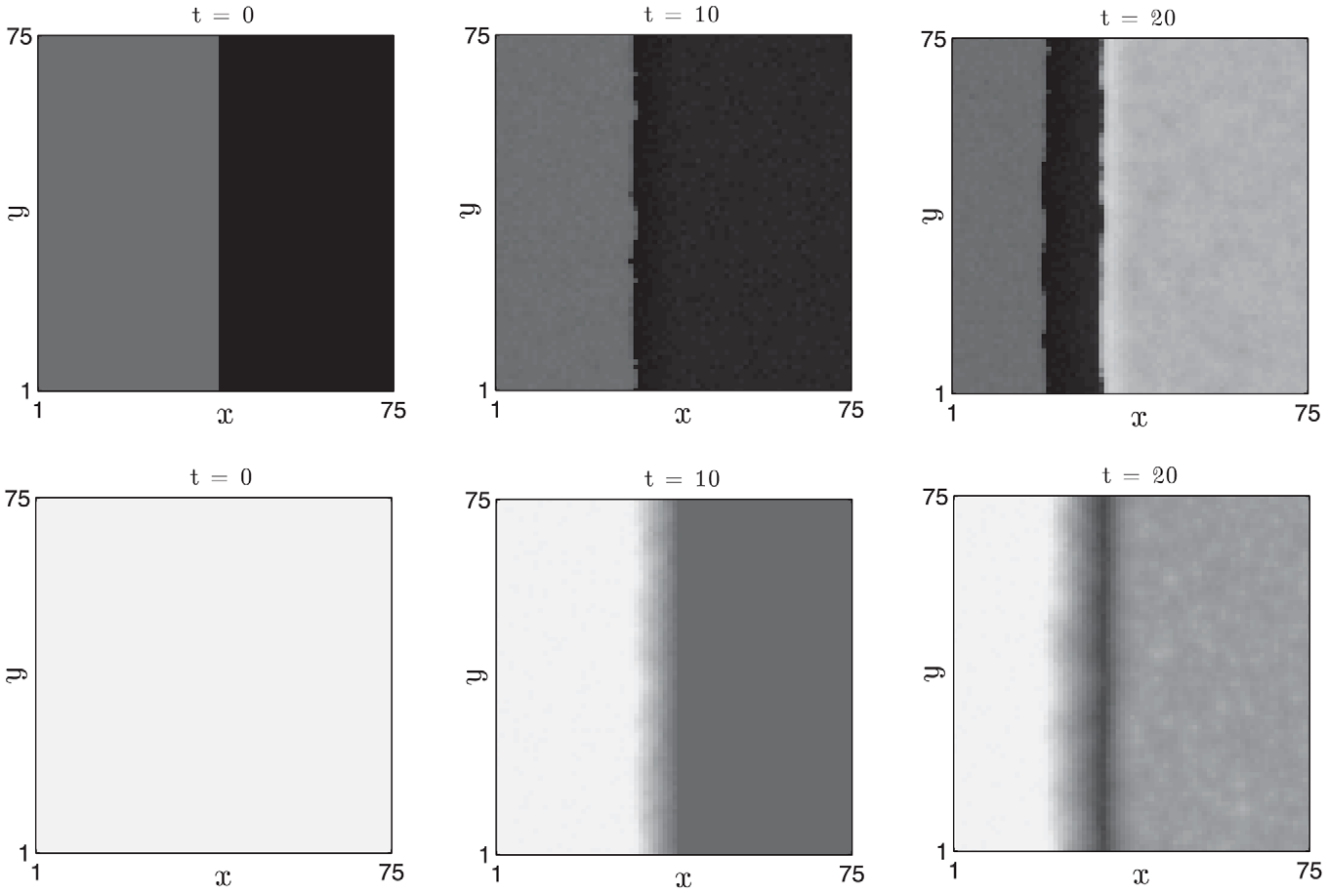
Border instability when the initial data are chosen such that the left- and right- hand sides of the domain are in competent and excited phases, respectively. Other details as in Figure 10.

In contrast, if the spatial domain is initialised such that the follicles on the left- and right- hand sides are in competent and excited phases, respectively, the excited follicles excite their immediate neighbours and the initial border between competent and excited follicles propagates into the competent domain (see Figure 12).

The simulation results presented to date have shown that the governing excitable medium equations produce phenomena at the individual follicle and follicle population scales that are consistent with the previous PARC model and experimental observations. We now investigate how our model can be applied to perturbed experimental systems, such as the *KRT14-Wnt7a* and *KRT14-Nog* mutant mice.

### Simulating perturbed systems

#### Bead experiments

**Figure 13:**
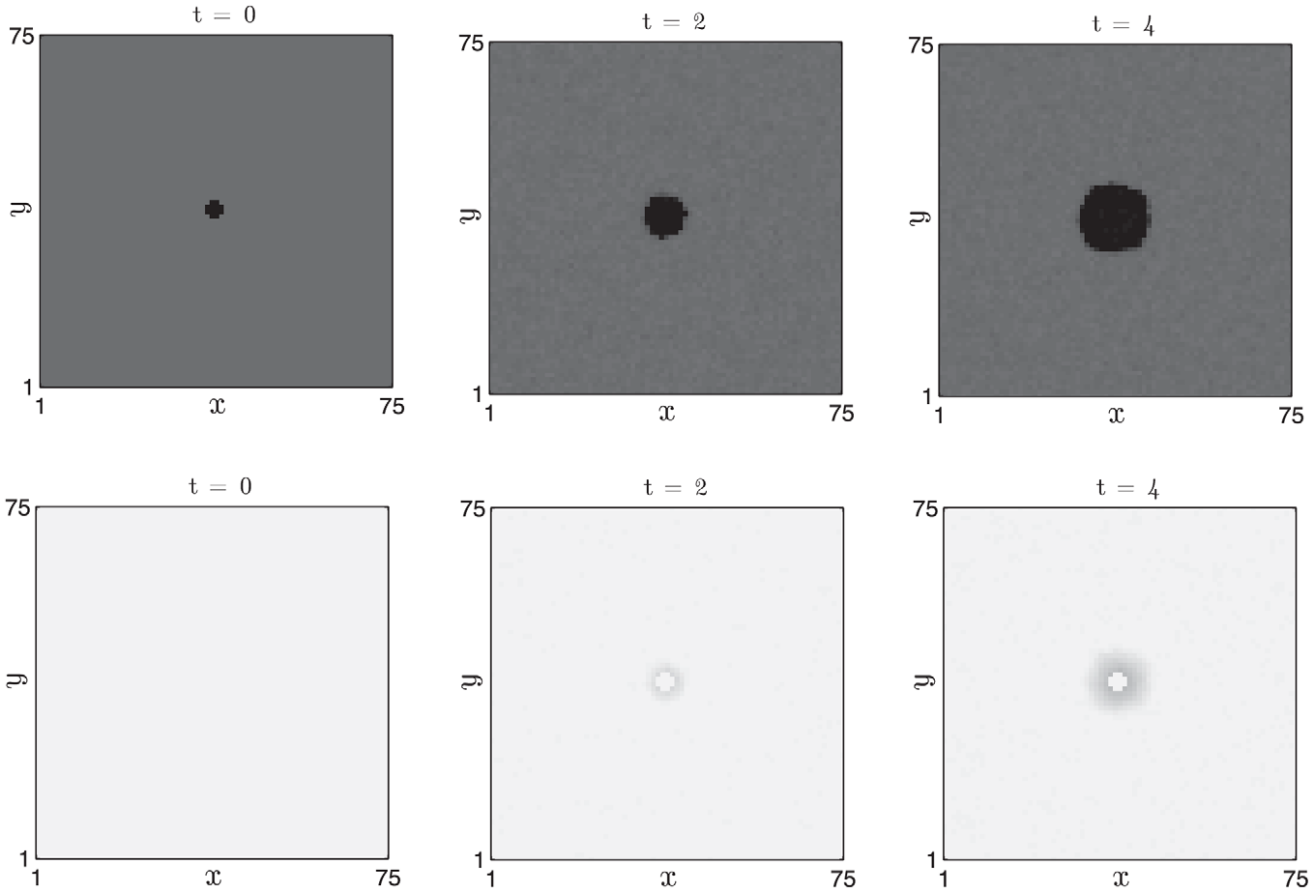
Simulating the presence of an activator-coated bead at the centre of the domain. The bead is approximated by fixing the activator and inhibitor activities such that 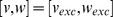 (see Text S1 for definitions) in the spatial domain 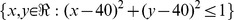. Other details as in Figure 10.

The implantation of activator- and inhibitor-coated beads into the surface of the skin results in local regions of follicle growth and growth retardation, respectively [30]. In Figure 13 we present results from a simulation in which the presence of an activator-coated bead is approximated by fixing the activities of the variables 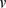 and 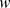 to the values of an excited follicle (see parameters 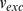 and 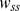 in Text S1) in a region of unit radius at the centre of the spatial domain. As expected, we observe that the effect of the activating bead is to force nearby follicles to become activated.

#### The *KRT14-Wnt7a* over-expression mouse

Having developed a model that allows us to relate a molecular description of follicle behaviour with macroscale observations of follicle growth, we now demonstrate the merit of this approach by investigating if changes to the molecular parameters can yield phenotypes observed in perturbed mouse models.

We begin by summarising the experimental observations made by Plikus et al. [16] with respect to the *KRT14-Wnt7a* over-expression mouse. At the molecular scale, the Wnt7a gene was constitutively expressed, thus periodic expression of this activator was replaced by constitutive expression. At the individual follicle scale, the time spent in anagen was relatively unchanged but the refractory and competent telogen times were significantly reduced relative to wild-type mice (see Table 1). At the population scale, waves of anagen propagation travelled at greater speeds and the rate of spontaneous activation was increased relative to wild-type mice. Moreover, target-like patterns with outwardly propagating rings of anagen were observed (see Figure 4 and [16]). It is not currently understood what mechanisms drive the emergence of target patterns at the population scale, nor why they are not always observed.

**Figure 14:**
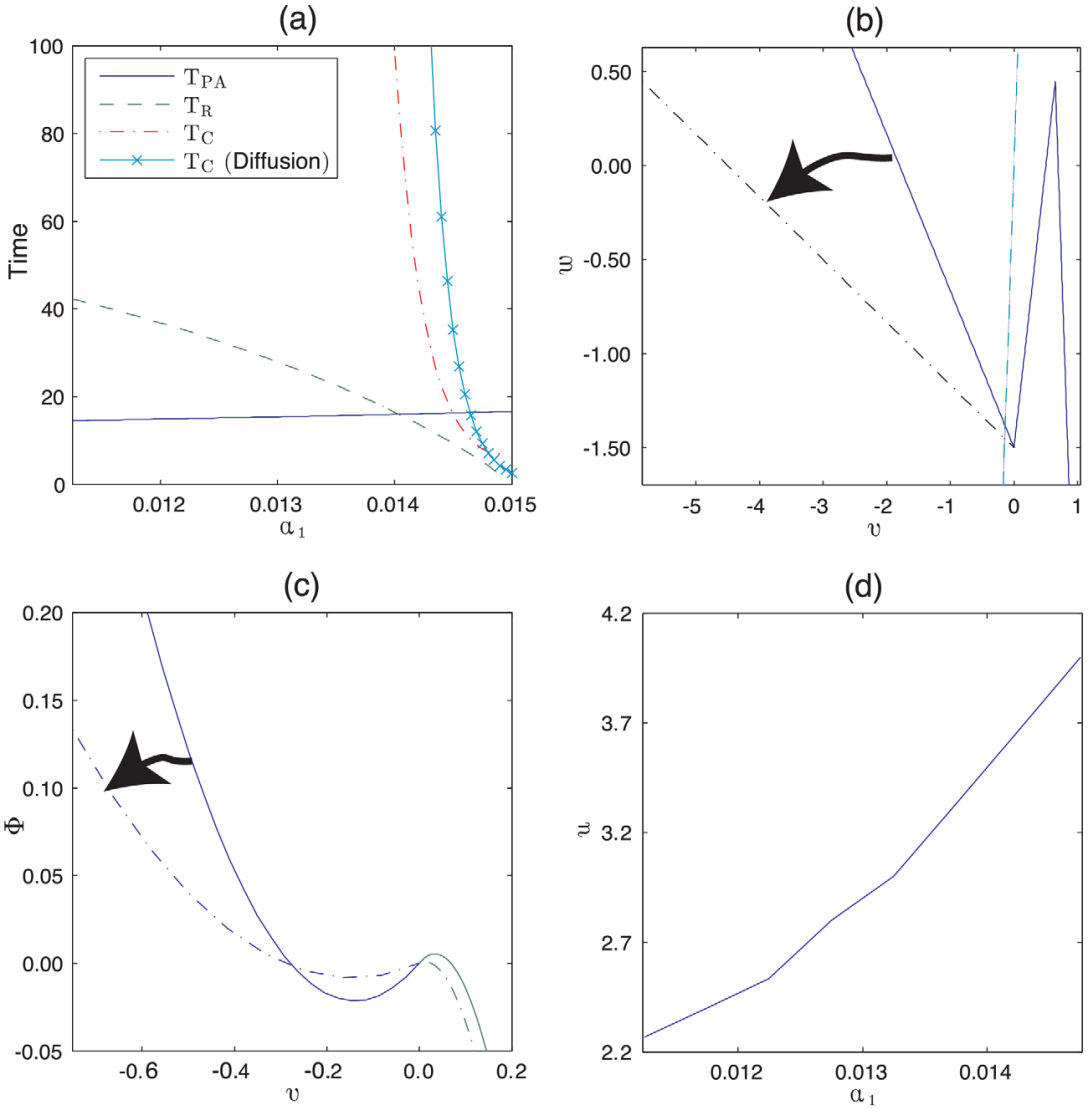
Variation of macroscale measurables with the activator production rate 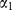
 **.** (a) The times spent in anagen and refractory and competent telogen (calculated using equations (4), (5) and (8)) are plotted against 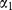. Note that the competent telogen time that accounts for diffusive coupling (see Text S1 for derivation) to nearest neighbours (solid line with crosses) is scaled by a factor of 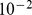 for plotting purposes. (b) The 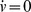 nullclines are plotted near the steady-state for both wild-type (solid line) and mutant (dot-dashed line) values of 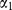. The dashed line denotes the fixed 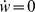 nullcline. (c) The effective potential energy, 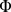, (introduced in equation (8) and derived in Text S1) is plotted against activator activity, 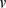. The shallower potential well for the mutant parameters (dot-dashed line) explains why 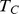 decreases with increasing 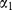. (d) The wave velocity, 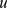, (calculated using a numerical solution of equations (12) and (13)) is plotted against 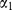. Arrowheads in (b) and (c) depict the effect of increasing 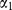.

It is notable that Wnt7a over-expression does not result in the destruction of the hair follicle cycle, as one might expect if Wnt7a was the only oscillating activator in the system. Rather, it perturbs quantitative features of the hair follicle system but the functional states defined by the P, A, R and C phases remain intact (Plikus et al. [16] have measured the lengths of the respective phases). This observation immediately informs us that there is not a linear relationship between Wnt7a concentration and the activator in our model described by the variable 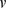. However, utilising the derived relationships (4), (5) and (8), we find that increasing positive feedback in the activator pathway (increasing 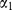) yields decreased competent and refractory times whilst anagen times remain approximately unchanged (see Figure 14 (a)). The increased activator production rate results in an increased rate of degradation of inhibitor in the refractory phase (see modified nullcline in Figure 14 (b)) and thus a shorter refractory phase. Meanwhile, the time spent in competent phase is reduced as increasing 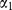 causes the steady-state to become less stable to stochastic fluctuations (see Figure 14 (c)). Hence, increasing the activator production rate results in the experimental observations of relatively unchanged anagen time and reduced competent and refractory telogen times.

**Figure 15:**
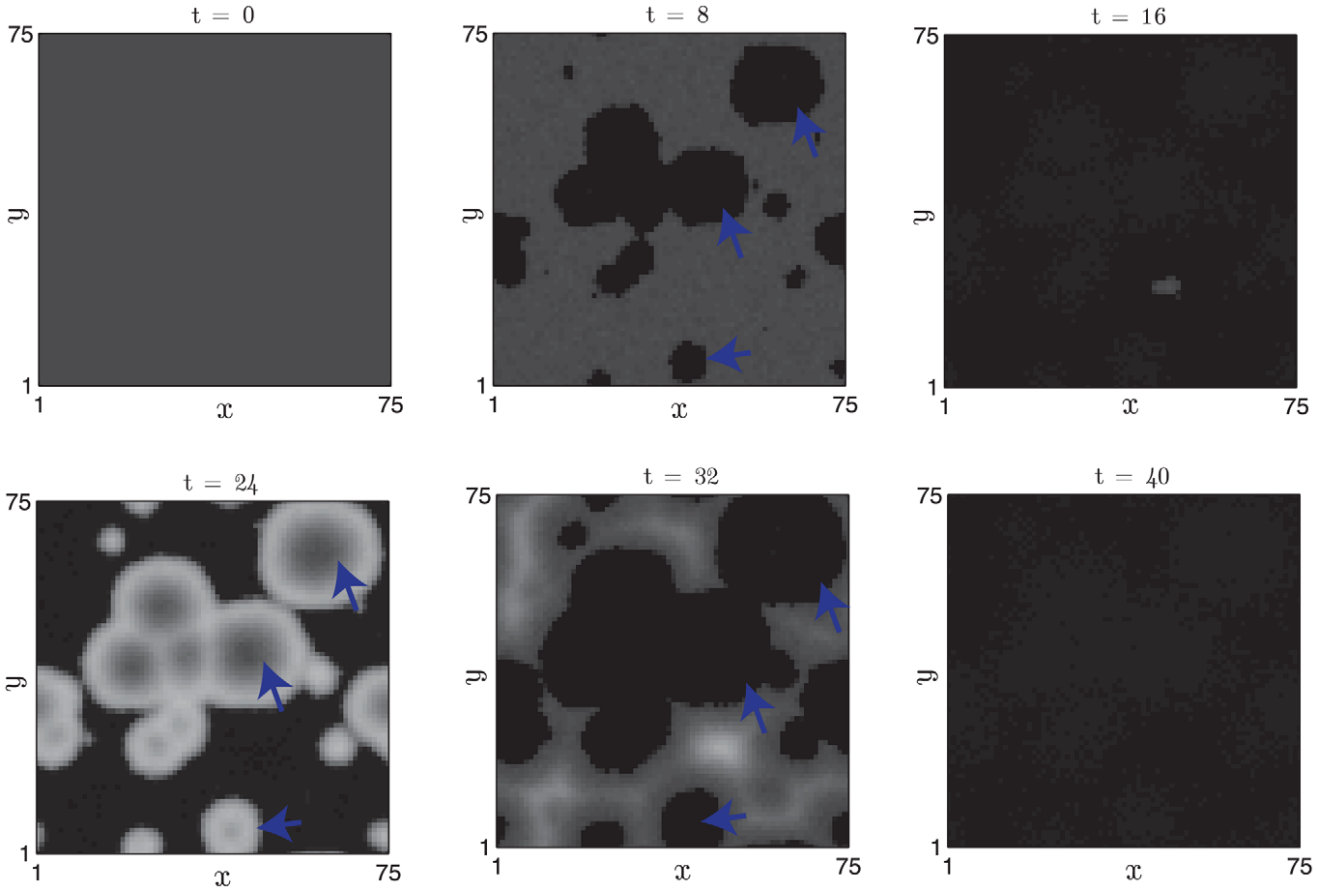
An increased rate of spontaneous initiation, faster propagation wavespeeds and target-like patterns arise upon increase of the parameter 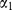 (compare with Figure 10). Activator activities (white - low, black - high) are plotted at 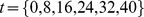 days. Equations (12) and (13) were solved with periodic boundary conditions. Arrowheads denote selected spontaneous activation sites that become re-excited in (e). 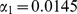 and other parameter values as in Table 2. For these parameters: 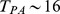 days, 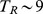 and 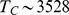 days.

Whilst it is encouraging that an intuitive increase in the activator production rate can recapitulate experimental observations at the single follicle scale in the Wnt7a over-expression mouse, we can use the multi follicle model to predict population scale phenomena and then compare results with the experimental observations. We find that the increased positive feedback rate yields an increased activation front wave speed (see Figure 14 (d)) and an increased rate of spontaneous initiations (compare Figures 10 and 15). Moreover, increasing the positive feedback results in the emergence of target patterns (see Figure 15 and Figure S3 in Text S1). Notably, the target patterns arise as a result of diffusion-mediated inter-follicle interactions occurring over multi follicle length scales; a follicle at the centre of a target pattern that is exiting refractory telogen has greater activator activity than follicles in its immediate locality (which are still in refractory telogen) resulting in a local activator maximum (and hence a negative diffusion term) that then causes a destabilisation of the excitable steady-state and anagen re-entry.

In summary, increased positive feedback in the activator dynamics results in the observed phenomena of faster activation wavefronts, shorter refractory and competent telogen times, unchanged anagen time, increased spontaneous initiation rates and the emergence of target patterns at the population scale. From these observations we propose that a likely role for Wnt7a expression is to increase positive feedback amongst the different activators of hair follicle growth.

#### The *KRT14-Nog* over-expression mouse

In a similar manner to the previous section, we investigate if the proposed description of follicle excitability can be used to lend insight into observed behaviour in the *KRT14-Nog* over-expression mouse. We begin again by summarising the experimental observations made by Plikus et al. [30]. At the molecular scale, the BMP inhibitor Noggin is constitutively over-expressed and thus its periodic expression is lost. At the single follicle scale, a dramatic decrease in the total length of time spent in telogen (*i.e.* refractory and competent phases) is observed (see Table 1) whilst anagen times are approximately unchanged. At the multi follicle scale, ‘simplified’ and ‘dynamic’ hair growth domains and ‘continuous wave propagation’ are observed (see Figure 4). We highlight a striking feature of the *KRT14-Nog* mouse: although competent telogen is much shorter than in wild-type mice, stochastic excitations are not observed at the multi follicle scale. It is not well understood what mechanisms of communication allow follicles to synchronise over the longer length scales, nor how observations at the different scales are interrelated.

As Noggin over-expression does not destroy the hair follicle cycle (anagen and telogen are still well defined), we again note that the activities of activators and inhibitors in the model are not explicitly representative of the expression patterns of individual genes. But can the observations at the follicle and multi follicle scales be interpreted in the proposed excitable medium framework? A striking observation in the *KRT14-Nog* mouse is the significantly reduced time spent in competent telogen (0–5 days) as compared with wild-type mice. As Noggin is an inhibitor of BMP, which is itself an inhibitor of hair follicle growth, we postulate that Noggin over-expression results in decreased inhibitor activity in the model. Notably, a sufficiently large decrease in the parameter 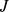 results in a qualitative change in follicle activity from excitable to oscillatory with the result being that competent telogen is lost.

**Figure 16:**
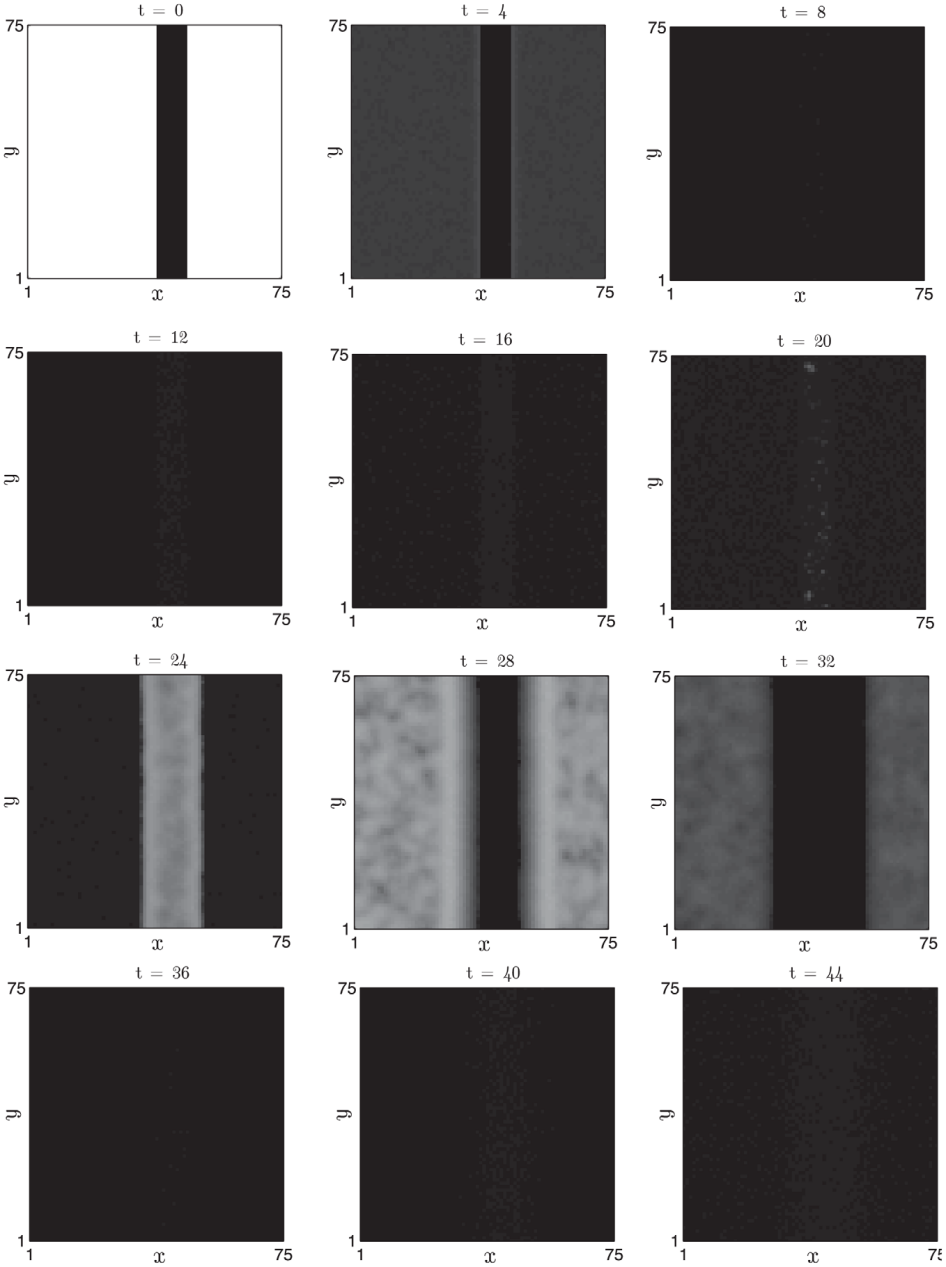
Oscillatory dynamics are observed upon decreasing the inhibitor production rate and increasing the activator production rate. Details as in Figure 15 with 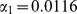 (increased by 5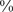) and 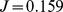 (decreased by 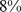). For these parameters: 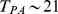 days and 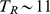 days.

 In order to illustrate population-scale behaviour when follicles are in the oscillatory regime, in Figure 16 we present simulation results from a field of oscillatory follicles (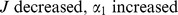) in which a central strip is initialised in anagen of the clock cycle whilst the other follicles are initially in refractory telogen. We observe that, over the time scale of observation, the initial border remains stable as the different regions of the spatial domain oscillate out-of-phase with one another. We suggest that a perturbation which pushes the follicles into the oscillatory regime might result in the sort of ‘simplified’ patterns observed in the *KRT14-Nog* mouse, as phenomena associated with excitability are lost. In the oscillatory regime we highlight that the ‘dynamic’ hair growth domains and ‘continuous wave propagation’ observed in the *KRT14-Nog* mouse could arise as a result of a qualitative change in follicle activity mediated by reduced inhibitor activity occurring as a consequence of Noggin over-expression.

 The motivation for a decrease in the parameter 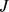 in the *KRT14-Nog* over-expression mouse is that Noggin is a BMP antagonist, thus a decrease in the parameter 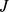 represents a reduced production of inhibitor as a result of the over-expression of its antagonist. However, a notable feature of the model is that a decrease the parameter 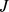 does not yield the reduced refractory telogen times observed by Plikus et al. [30]. In order to recapitulate this phenomenon we assume that an effect of Noggin over-expression is to simultaneously increase positive feedback in the activator pathway (i.e the parameter 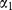 is also increased in a similar manner to the *KRT14-Wnt7a* over-expression mouse). This perturbation can be motivated by recalling that Kobielak et al. have shown that 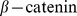 transcription is elevated in the presence of excessive Noggin [15]. In summary, a combination of increased positive back and a decreased inhibitor production rate results in reduced refractory and competent telogen times, the loss of the excitable steady-state and the emergence of oscillatory dynamics.

### Testing the excitable medium hypothesis

In this section we suggest a number of further experiments which could help to further determine the excitable properties of the hair follicle system.

#### Separation of timescales

**Figure 17:**
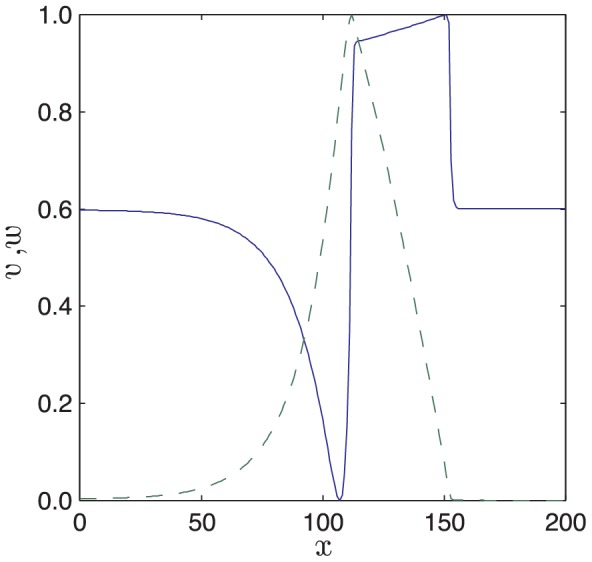
Separation of time scales yields qualitative difference in the spatial gradients of activator (solid line) and inhibitor (dashed line). A snapshot of a slice through the 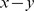 plane (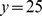) from the plane wave solution presented in Figure 12. Equations (12)–(13) were solved with parameter values as in Table 2.

A key unvalidated assumption in our model is that there is separation of time scales between activator and inhibitor dynamics. One testable effect of such time scale separation is that spatial gradients of the activator (fast variable) should be much larger than those of the inhibitor (slow variable). In Figure 17 we plot the activities of activators and inhibitors through a slice of the plane wave in the simulation results presented in Figure 12. Note the much sharper spatial gradients of the activator compared with the inhibitor.

#### Generation of spiral waves

**Figure 18:**
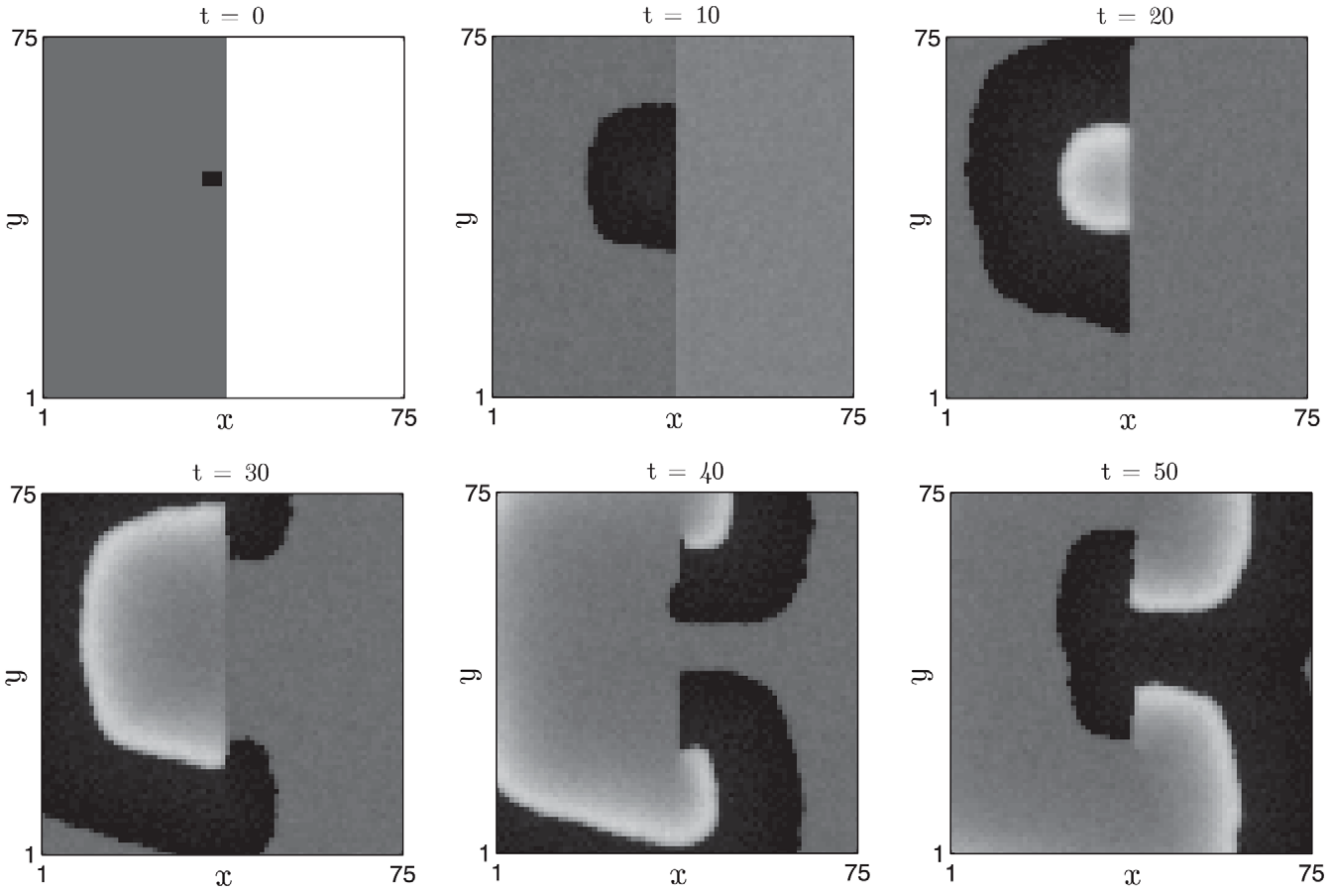
Transient spirals can be generated from heterogeneous initial data. The right-hand side of the domain is initially in refractory telogen, the left-hand side is in competent telogen and a small region centred at 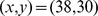 is in propagating anagen. Activator, 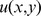 activity (white - low, black - high) is plotted at 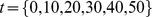 days. Equations (12)–(13) were solved with parameter values as in Table 2.

If the hair follicle system is truly an excitable medium, it should be possible to conduct experiments in which spiral waves, a ubiquitous feature of excitable medium dynamics, are induced. One way in which to do this is to introduce spatial inhomogeneity into the excitable medium and, in our numerical simulations, this can be achieved using the initial data presented in Figure 11 in which the spatial domain is initially subdivided into competent and refractory regions and a small excitation is induced near the border. The initial excitation first spreads through the competent region but cannot excite the refractory follicles. Eventually, as depicted in Figure 18, the refractory region returns to competent telogen but becomes excited by the spreading anagen wave in a manner that can generate spiral-like patterns of activation.

#### Thresholding

**Figure 19:**
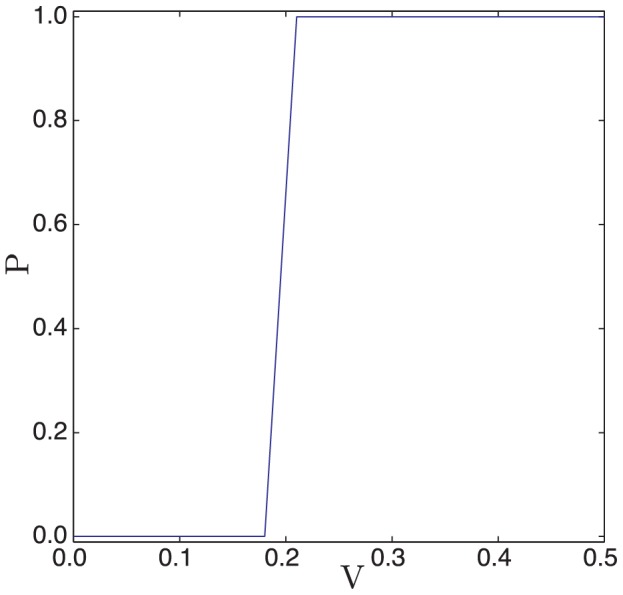
The determination of an activation threshold for a bead placed at the centre of the spatial domain. The probability that the bead activates neighbouring follicles, 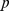, is plotted against the activity of the implanted bead, 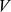. Equations (12)–(13) were solved with parameter values as in Table 2.

A defining characteristic of an excitable medium is the phenomenon of thresholding, *i.e.* a follicle should have a stable steady-state from which a characteristic perturbation is required for it to become excited. Although the property of an activity threshold has previously been highlighted [1], if the follicle field is an excitable medium it should be possible to find the threshold experimentally, potentially via the implantation of beads with controlled activator concentrations. In such a scenario, one would expect that an implanted bead has no effect and causes local anagen initiation events at low and high activator concentrations, respectively. Moreover, there should exist some intermediate threshold at which the system switches between behaviours. We have conducted numerical simulations in which a bead with different levels of activator activity is simulated and measured whether or not it results in the excitement of neighbouring follicles. In Figure 19, where we plot the probability of excitement against the activator activity on the bead, it is clear that there is a critical threshold above which the bead excites neighbouring follicles.

## Discussion

Recent experimental work in the hair follicle system has allowed the gathering of information across a range of spatial scales: at the molecular scale, numerous pathways have been shown to activate and inhibit follicle growth; at the single follicle scale, hair plucking assays have allowed quantification of the time spent in the different phases of the follicle cycle; and at the multi follicle scale, hair clipping assays have allowed the characterisation of population scale behaviours, such as wave propagation. The interdependence between the different scales is only beginning to become understood.

A previous model of mouse hair follicle growth proposed by Plikus et al. related the individual and multi follicle population scales [11], [16]. The hair plucking assay data were used to parameterise the PARC model and the simulation of populations of follicles allowed investigation of the interplay between the characteristic times spent in the different phases of the clock cycle and emergent patterns. Similarly, Halloy et al. [17], [18] previously considered a stochastic follicle automaton model of hair growth in humans. Notably, human hair growth patterns do not exhibit the same wave-like growth patterns as mice and it is thought that inter-follicular coupling is either not present or at least much weaker than in mice. Given recent advances in understanding of the molecular regulators of hair follicle growth, a disadvantage with cellular automaton frameworks is that it is not obvious how to relate the PARC phase times to observations at the molecular scale.

In this study we propose a stochastic, two-variable, activator-inhibitor model of mouse hair follicle growth dynamics. An important feature of the model is that the functional phases of the hair follicle cycle are emergent, thus allowing us to relate hair plucking measurements at the single follicle scale to underlying molecular regulation. Whilst the two-variable description of molecular events is undoubtedly an abstraction, we believe it is justified in the present case for the following reasons: (a) although the molecular pathways underlying follicle growth are becoming increasingly better understood, the current level of description is qualitative at the molecular scale, making the parameterisation of detailed molecular models difficult; (b) the model is tractable and can thus help to develop insight into how measured effects at different spatial scales are inter-related; and (c) the model can be formulated in a manner allowing comparison with both previous models and experimental observations. We anticipate that increasing quantification at the molecular scale will enable our description of underlying molecular interactions to be fine-tuned in future iterations of the model.

After developing an excitable, stochastic model of a single follicle, we considered a two-dimensional field of diffusively-coupled follicles, as previously suggested by Plikus et al. [11]. Model simulations exhibited many features in common with experimental observations: activator-inhibitor dynamics in qualitative agreement with known activators and inhibitors of follicle growth; stochastic, spontaneous initiations causing a single follicle to pass through the excitability threshold; propagation through the excitable medium of single waves originating from a single excitation; border stability when an excitation occurs close to a refractory region; border instability when a border separates a region of excited and competent follicles; and the emergence of regions of localised activation upon simulation of an activator-coated bead.

We propose that an advantage of the current framework is that it allows one to investigate how changes at the molecular scale might give rise to different patterning phenotypes. In the *KRT14-Wnt7a* mouse, the activator Wnt7a is constitutively over-expressed and Plikus et al. observed decreased refractory and competent phase times, an increased spontaneous initiation rate, faster excitation waves and the emergence of target-like patterns. Notably, the constitutive over-expression of Wnt7a did not destroy the follicle cycle as the different functional phases were still distinguishable. To the best of our knowledge, there is no well-understood mechanism describing why the observed patterns arise in this particular mutant. We set about trying to investigate the *KRT14-Wnt7a* phenotype within the proposed framework and found that an increase in the activator positive feedback strength resulted in decreased refractory and competent telogen times at the follicle scale. We then investigated the effect of the increased production rate at the follicle population scale and found an increased wave velocity and greater propensity for stochastic excitations. Intriguingly, the population scale patterns changed from being single waves of excitation to target patterns. Notably, the target patterns arise as a consequence of diffusive coupling acting over multiple follicles, a behaviour that represents a significant deviation from the previous PARC model, where coupling only occurred between neighbouring competent and anagen follicles. Furthermore, we have demonstrated that coincident increased activator and decreased inhibitor production rates yield a shorter refractory phase and an oscillatory follicle, and have suggested that such a change in follicle stability might be responsible for population scale observations in the *KRT14-Nog* mouse.

A notable feature of our simulations is that competent telogen times must be of the order of 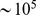 days such that the frequency of stochastic excitations across a population of follicles is comparable to the population scale patterns measured by Plikus et al. However, at the single follicle scale Plikus et al. have measured competent telogen times in the range of 0–60 days. In fact, when we used these much shorter competent telogen times the simulations are dominated by stochastic excitations in a manner inconsistent with population scale measurements from wild-type mice (data not shown). In order to resolve this apparent conflict we highlight that: (a) the mouse system is dominated by the nearest-neighbour propagation mechanism of anagen initiation, thus placing an upper bound on the observation range for stochastic excitations (i.e. after 60 days a propagating wave has excited a given follicle, thus biasing the observation range of stochastic excitation events); and (b) the proposed model predicts an exponential distribution of competent telogen times, hence it is exponentially less likely to observe longer competent telogen times than shorter ones. In summary, viewed through the theoretical framework proposed in this study, competent telogen in mice should be much more stable than one might immediately infer from previous measurements. This prediction could be investigated in experimental work in which the inter-follicular communication mechanism is disrupted.

The model presented in this study has a number of limitations. Firstly, we do not have direct estimates of molecular parameters, such as decay rates and cross-activation and -inhibition rates of activators and inhibitors. Secondly, we note that *in vivo* geometries have both periodicity and boundary effects that might influence emergent patterns in real systems. Thirdly, in the mouse mutants the time spent in anagen does not vary while in our model this depends strongly on parameters such as the inhibitor production rate (data not shown). Fourthly, diffusion and stochastic effects have been modelled only for the activator dynamics. These details could also be introduced into the inhibitor dynamics but at the expense of further model complication. Finally, our model does not explicitly account for biophysical changes that occur in a follicle as the hair cycle progresses or sub-follicular structures such as dermal papillae. However, whilst it will be important to account for the aforementioned limitations in future iterations of the model, it is our belief that the central thesis of this study, that populations of hair follicles can be treated as an excited medium, will remain unchanged once these limitations have been addressed. We envisage that in the same manner as the Fitzhugh-Nagumo equations can be used as a caricature description of the dynamics of action potential propagation in cardiac tissue, the model proposed in this study might provide a caricature description of hair follicle growth propagation.

Before the proposed model is embellished to account for further details of underlying hair follicle biology, there are a number of conceptually simpler experiments that could allow us to further validate the central thesis of this study. Firstly, the separation of time scales in the model allows a clear distinction between excited and competent phases of the cycle. In our model, the activator changes on a much faster time scale than the inhibitor and this is observable by much larger spatial gradients in the activators. Secondly, a ubiquitous feature of excitable media is the presence of spiral waves. If the hair follicle system is an excitable medium, one would expect that particular initiations of oscillators would result in the development of propagating spirals. Finally, excitable media typically exhibit a thresholding property whereby a stimulus of a sufficiently large magnitude is required to excite a given follicle. Hence, one would expect that such a threshold could be identified by examining the behaviour of beads coated with different activator concentrations.

On a concluding note, the regulation of regeneration and renewal is a key characteristic of any homeostatic biological system. In this study we have coupled hair follicle growth to the activity of activator in an excitable medium, a hypothesis that seems particularly attractive given that growth occurs only on a transient time scale. We expect that if further substantiated in the hair follicle system, there may be other instances where an excitable medium framework can be used as a mechanism for regulating regeneration in homeostatic systems.

## Supporting Information

Text S1 **Derivation of the times spent in anagen, refractory telogen and competent telogen.**
(PDF)
