# Supplementary material for: Modelling Hair Follicle Growth Dynamics as an Excitable Medium
Source: PLoS Comput Biol. 2012 Dec 20;8(12):e1002804. doi: 10.1371/journal.pcbi.1002804 (PMC3527291; doi:10.1371/journal.pcbi.1002804)
Supplement: Text S1 — Derivation of the times spent in anagen, refractory telogen and competent telogen. (PDF) [file pcbi.1002804.s001.pdf]

Supplementary Material: Modelling hair follicle growth dynamics as an excitable medium

Philip J. Murray[1*], Philip K. Maini[1,2], Maksim V. Plikus[3] Cheng-Ming Chuong[4] Ruth E. Baker[1]

1 Centre for Mathematical Biology, Mathematical Institute, 24-29 St Giles', Oxford, OX1 3LB, UK

2 Oxford Centre for Integrative Systems Biology, Department of Biochemistry, South Parks Road, Oxford, OX1 3QU, UK

3 Department of Dermatology, University of Pennsylvania, Philadelphia, PA 19104, USA

4 Department of Pathology, University of Southern California, Los Angeles, CA 90033, USA

∗ **E-mail: murrayp@maths.ox.ac.uk (corresponding author)**

1 Model analysis

The dynamics of equations (1) – (2) can be understood by considering the nullclines (lines in phase space where $\dot{v}$ and $\dot{w}$ are zero, respectively), which are given by the equations

$$w = \frac{f(v) - av + I}{b}; \tag{1}$$

$$w = \frac{cv + J}{d}. \tag{2}$$

with f given by equation (3) in the main text.

We define the following quantities (see Figure 1):

$$v_{ss} = \frac{Id - Jb}{d(a - \alpha_1) + cb}, \tag{3}$$

$$w_{ss} = \frac{Ic + J(a - \alpha_1)}{d(a - \alpha_1) + cb}, \tag{4}$$

$$w_{max} = \frac{\alpha_1 v_0 + \alpha_2 (v_1 - v_0) - a v_1 + I}{b}, \tag{5}$$

$$v_i = \frac{d(\alpha_1 v_0 + \alpha_2 (v_1 - v_0) + I) - bJ}{cb + ad}, \tag{6}$$

$$v_{min} = \frac{I - b w_{max}}{a - \alpha_1}, \tag{7}$$

$$v_{thr} = \frac{(\alpha_2 - \alpha_1) v_0 + b w_{ss} - I}{\alpha_2 - a}, \tag{8}$$

$$v_{max} = \frac{\alpha_1 v_0 + \alpha_2 (v_1 - v_0) - b w_{ss} + I}{a}, \tag{9}$$

where v_{ss} and w_{ss} denote the stable steady state activator and inhibitor activities, respectively, w_{max} denotes the maximum inhibitor activity (as $\epsilon \to 0$), v_i denotes the intersection point of the w nullcline with the extended right-most part of the v nullcline, v_{min} denotes the minimum activator activity (as $\epsilon \to 0$) and v_{thr} denotes the threshold in v that must be overcome in order for a follicle to become excited. A follicle is in the excitable regime when the following conditions hold:

$$\alpha_1 < a < \alpha_2, \tag{10}$$

$$v_{min} < v_{ss} < v_0, \tag{11}$$

$$v_{ss} < v_i < v_1. \tag{12}$$

1.1 Calculating the time spent in anagen and refractory telogen

A piecewise linear approximation to the activator production dynamics allows us to derive estimates for quantities that can be related to the PARC model. For instance, on the upwards portion of the excitable trajectory we approximate that the fast variable is in pseudo-equilibrium, hence

$$\epsilon \dot{v} \sim 0, \tag{13}$$

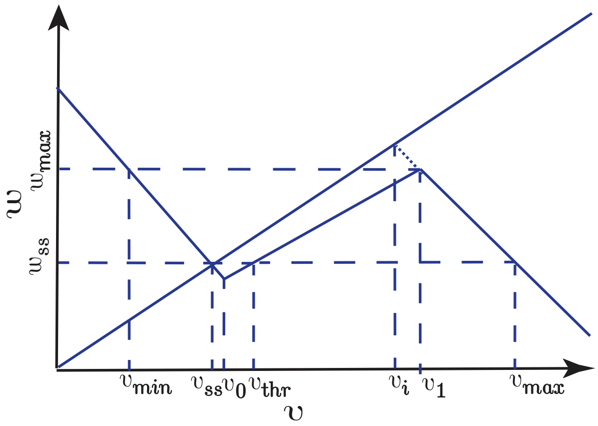

Figure S1. A schematic illustration of notable activator and inhibitor activities.

and the activator activity can be approximated using

$$v \sim \frac{\alpha_2(v_1 - v_0) + \alpha_1 v_0 - bw + I}{a}. \tag{14}$$

An ordinary differential equation for the inhibitor activity on the slow time scale is then given by

$$\dot{w} = -w\left(\frac{bc}{a} + d\right) + J + \frac{c}{a}\left(\alpha_1 v_0 + \alpha_2(v_1 - v_0) + I\right), \tag{15}$$

which can be integrated from the steady-state value of w to the maximal value, and an estimate for the time spent on the upwards portion of the trajectory is found to be

$$T_{PA} \sim \frac{a}{cb + ad} \ln\left(\frac{\frac{cI + J(a-\alpha_1)}{d(a-\alpha_1)+cb} - \frac{c(\alpha_1 v_0 + \alpha_2(v_1 - v_0) + I) + Ja}{da+cb}}{\frac{\alpha_1 v_0 + \alpha_2(v_1 - v_0) - av_1 + I}{b} - \frac{c(\alpha_1 v_0 + \alpha_2(v_1 - v_0) + I) + Ja}{da+cb}}\right). \tag{16}$$

Similarly, the time spent on the downwards nullcline is

$$T_R \sim \frac{(a - \alpha_1)}{cb + d(a - \alpha_1)} \ln\left(\frac{\frac{\alpha_1 v_0 + \alpha_2(v_1 - v_0) - av_1 + I}{b} - \frac{cI + J(a-\alpha_1)}{cb + d(a-\alpha_1)}}{\delta_w}\right), \tag{17}$$

where δ_w represents a characteristic displacement of w from steady-state (see below).

1.2 Calculating the time spent in competent telogen

As the system is in the excitable regime, it has a single steady-state which we define to be (v_{ss}, w_{ss}). Assuming ϵ is sufficiently small such that the noise-modified dynamics near the steady-state are governed by the fast-time scale equations [1, 2], we approximate that

$$\begin{aligned} \epsilon\dot{v} &= f(v) - av - bw + I + \xi(t), \\ w &\sim w_{ss}. \end{aligned} \tag{18}$$

Hence, equations (19) can be written in the form

$$\dot{v} = -\nabla\Phi + \frac{\xi(t)}{\epsilon}, \tag{19}$$

where the potential energy function, Φ, is given by

$$\Phi = -\frac{1}{\epsilon}\int^{v}\left[f(v') - av' - bw_{ss} + I\right]dv'. \tag{20}$$

When f is piece-wise linear Φ takes the form

$$\Phi(v) = \begin{cases} \frac{(a-\alpha_1)}{\epsilon}(\frac{v^2}{2} - v_{ss}v) + C_1 & \text{if } v < v_0, \\ \frac{(a-\alpha_2)}{\epsilon}(\frac{v^2}{2} - v_{thr}v) + C_2 & \text{if } v_0 < v < v_1, \end{cases} \tag{21}$$

where C_1 and C_2 are integration constants chosen such that Φ is continuous across the internal boundaries.

Near the steady-state of the excitable system, Φ takes the form of a bistable potential well (*e.g.* see Figure 14 (c)) and we can estimate the mean time for a particle to escape from the stable steady-state in the low noise limit using Kramers formula [3]:

$$T_{exc} = \frac{2\pi}{\sqrt{-\Phi''(v_{ss})\Phi''(v_{thr})}}\exp\left(-\frac{(\Phi(v_{thr}) - \Phi(v_{ss}))\epsilon^2}{\Gamma}\right). \tag{22}$$

In the case where a follicle is coupled to its neighbours via diffusion, the estimate for the mean time spent in competent telogen changes. We assume that a given follicle's neighbours are fixed at the steady-state, such that $\{v, w\} = \{v_{ss}, w_{ss}\}$ and the follicle is in competent telogen. The threshold value of v

beyond which the follicle becomes excited is found to be

$$v^*_{thr} = \frac{I + (\alpha_1 - \alpha_2)v_0 - bw_{ss} + nD_A v_{ss}}{a - \alpha_2 + nD_A}, \tag{23}$$

$$\tag{24}$$

where n is the number of nearest neighbours ($n = 4$) and D_A is the diffusion coefficient. The energy Φ is redefined to be

$$\bar{\Phi}(v) = \begin{cases} \frac{(a-\alpha_1)+nD_A}{\epsilon}(\frac{v^2}{2} - v_{ss}v) + C_1 & \text{if } v < v_0, \\ \frac{(a-\alpha_2)+nD_A}{\epsilon}(\frac{v^2}{2} - v^*_{thr}v) + C_2 & \text{if } v_0 < v < v_1, \end{cases} \tag{25}$$

and the mean time spent in competent telogen is

$$T^*_{exc} = \frac{2\pi}{\sqrt{-\bar{\Phi}''(v_{ss})\bar{\Phi}''(v^*_{thr})}} \exp\left(-\frac{(\bar{\Phi}(v^*_{thr}) - \bar{\Phi}(v_{ss}))\epsilon^2}{\Gamma}\right). \tag{26}$$

As one might expect, diffusive coupling of a given competent telogen follicle to neighbours also in competent telogen further stabilises the steady-state and the mean escape time increases.

1.3 The boundary between refractory and competent telogen

A one-dimensional approximation to the fast dynamics near the stable steady state is given by

$$\epsilon\dot{v} = -\gamma(v - v_{ss}) + \xi(t), \tag{27}$$

where $\gamma = a - \alpha_1$, v_{ss} is the stable steady-state with the noise term defined as in equation (6) in the main text. Notably, the noise perturbs the dynamics away from the stable steady state and the standard deviation of the trajectory is

$$\delta_v = \sqrt{\langle (v - v_0)^2 \rangle} = \sqrt{\frac{\Gamma}{2\gamma\epsilon}}, \tag{28}$$

where $\langle . \rangle$ denotes the mean value.

We use the standard deviation of the fast variable as a means of defining the end of refractory telogen

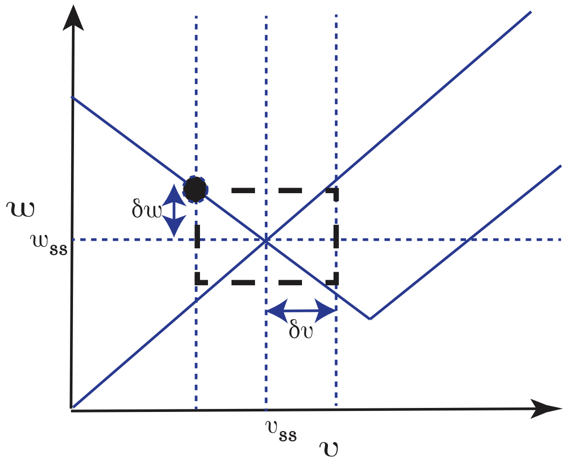

Figure S2. A schematic illustration of the defined boundary between refractory and competent phases. Null-clines are denoted by solid lines. δv denotes the standard-deviation of the stochastic fast variable in the one dimensional near steady state approximation. The dashed line box denotes a boundary in phase space where competent telogen is defined. Hence as a follicle moves through refractory telogen (large arrow), it reaches the dashed box approximately at the large dot and we assume that the stochastic dynamics of the fast variable provide a good approximation to the follicles dynamics.

such that when the fast variable is within a standard deviation of the steady state on the downwards nullcline, refractory telogen ends. Using the nullcline

$$w = \frac{(\alpha_1 - a)v + I}{b}, \tag{29}$$

we define (see Figure S2)

$$\delta_w = -\frac{(\alpha_1 - a)\delta v}{b} = \frac{1}{b}\sqrt{\frac{\Gamma(a - \alpha_1)}{\epsilon}}, \tag{30}$$

such that competent telogen is defined in the region $\{v, w : v > v_{ss} - \delta v, v < v_{ss} + \delta v, w > w_{ss} - \delta w, w < w_{ss} + \delta w\}$. Hence on the descending nullcline we only integrate the w ODE to within δ_w of the steady-state value and obtain that the time spent on this part of the trajectory is given by equation (17).

1.4 The emergence of target patterns in the deterministic model

In this section we set the noise strength $\Gamma = 0$ in order to examine the effect of increasing the parameter α_1. In Figure S3 we observe the propagation of single waves of hair growth. In Figure S4, where the parameter α_1 has been increased, a single excitation results in the the emergence of target patterns as well as larger front propagation speeds.

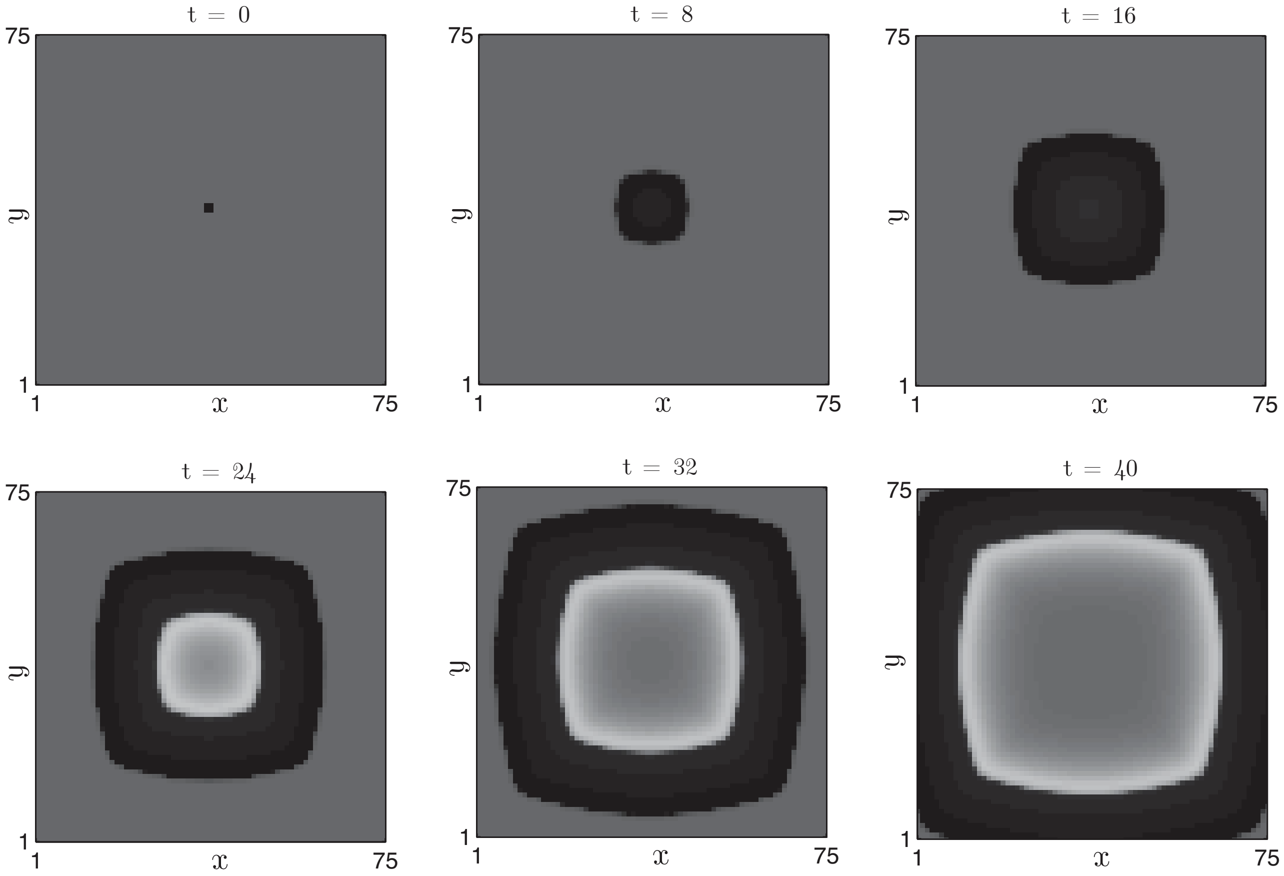

Figure S3. Propagation of a single wave in the deterministic limit ($\Gamma = 0$). Activator activities (white - low, black - high) are plotted at $t = \{0, 8, 16, 24, 32, 40\}$ days. Equations (12) and (13) from the Main Text were solved with periodic boundary conditions. $D_A = 3.25e^{-4}$ and other parameter values as in Table 2 from the Main Text.

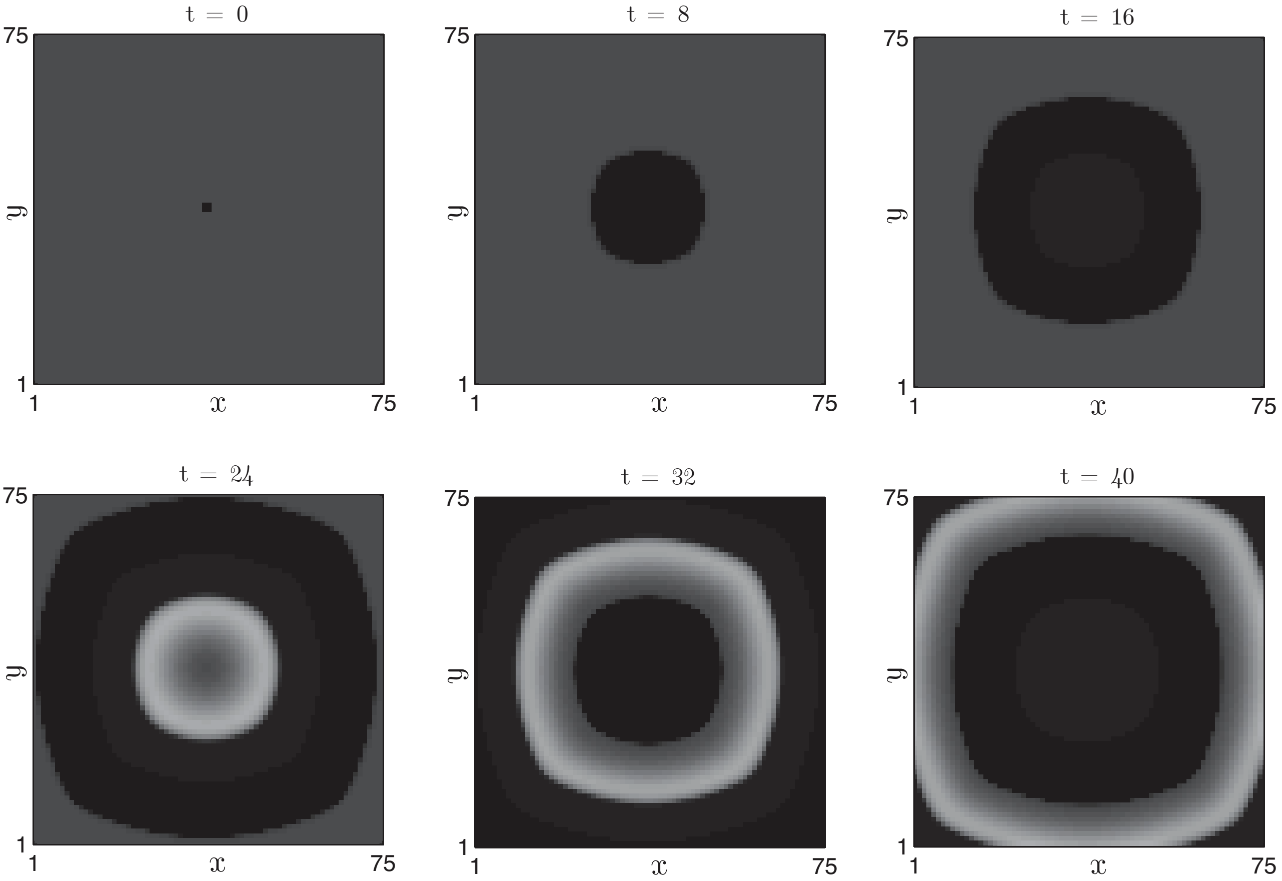

Figure S4. The emergence of target patterns and increased propagation speeds upon increase of the parameter α_1 (compare with Figure S3). Activator activities (white - low, black - high) are plotted at $t = \{0, 8, 16, 24, 32, 40\}$ days. Equations (12) and (13) from the Main Text were solved with periodic boundary conditions. $\alpha_1 = 0.0145$, $D_A = 3.25e^{-4}$, $\Gamma = 0$ and other parameter values as in Table 2 from the Main Text.

References

1. Collins JJ, Chow CC, Imhoff TT (1995) Aperiodic stochastic resonance in excitable systems. Phys Rev E 52: 3321–3324.

2. Tuckwell HC, Rodriguez R, Wan FYM (2003) Determination of firing times for the stochastic Fitzhugh-Nagumo neuronal model. Neural computation 15: 143–159.

3. van Kampen NG (1992) Stochastic Processes in Physics and Chemistry. North Holland.
